# Effects of aspirin and omega-3 fatty acids on age-related macular degeneration in ASCEND-Eye: a randomised placebo-controlled trial in a population with diabetes

**DOI:** 10.1136/bmjopen-2024-090605

**Published:** 2025-02-26

**Authors:** Emily Sammons, Louise Bowman, William Stevens, Georgina Buck, Imen Hammami, Sarah Parish, Jane Armitage, on behalf of the ASCEND-Eye Collaborative Group, Rory Collins

**Affiliations:** 1 Nuffield Department of Population Health, University of Oxford, Oxford, UK

**Keywords:** Clinical Trial, Ageing, Medical retina

## Abstract

**Purpose:**

Aspirin and omega-3 fatty acids (FAs) are potential disease modifiers of age-related macular degeneration (AMD), but previous studies have produced inconsistent findings. Randomised evidence for the efficacy and safety of aspirin and omega-3 FAs on AMD is presented in this study.

**Design:**

ASCEND-Eye is a substudy of eye effects in the 2×2 factorial design ASCEND (A Study of Cardiovascular Events iN Diabetes) double-blind, randomised, placebo-controlled trial for the primary prevention of cardiovascular events. Reports of AMD diagnoses were sourced from 6 monthly ASCEND follow-up questionnaires and a Visual Function Questionnaire.

**Participants:**

15 480 UK adults at least 40 years of age with diabetes but no evident cardiovascular disease.

**Interventions:**

100 mg aspirin daily versus placebo and, separately, 1 g omega-3 FAs daily versus placebo.

**Main outcome measure:**

The first post-randomisation reports of AMD.

**Results:**

During 7.4 years of follow-up, 122 (1.6%) participants randomised to aspirin were reported as having AMD, compared with 138 (1.8%) randomised to placebo (rate ratio 0.88; 95% CI 0.69 to 1.12; p=0.31). AMD occurred in 130 (1.7%) participants randomised to omega-3 FAs, compared with 130 (1.7%) randomised to placebo (rate ratio 0.99; 95% CI 0.78 to 1.27; p=0.99).

**Conclusion:**

No clinically-meaningful effects of aspirin or omega-3 FAs on AMD were found. Although the study had very limited statistical power to detect clinically relevant effects, these data overcome some methodological limitations of previous observational studies, providing randomised evidence of both treatments on AMD, which could contribute to future meta-analyses.

**Trial registration number:**

ISRCTN60635500 and NCT00135226.

**STRENGTHS AND LIMITATIONS OF THIS STUDY**

- The strengths of our study include its randomised design, well-defined exposure, a large number of participants and long duration of near-complete follow-up.
- ASCEND (A Study of Cardiovascular Events iN Diabetes)-Eye was underpowered to detect modest but plausible effects of each treatment. Therefore, it was not possible to compare the effects of aspirin or omega-3 fatty acids on prognostically important risk factors for age-related macular degeneration, including age and smoking.

## Introduction

 Age-related macular degeneration (AMD) is a leading cause of central visual loss in older people worldwide.^1^ Aspirin and omega-3 fatty acids (FAs) are potential modifiers of the disease process, but previous studies of their relationships with AMD have produced inconsistent findings. The long-term use of aspirin for more than 15 years has been associated with harmful effects on the wet form of AMD in some large observational studies^2 3^; however, two randomised trials reported non-significant trends towards a beneficial effect on their combined late AMD endpoint.^4 5^ Separately, observational studies have associated a higher consumption of oily fish or omega-3 FAs with a lower risk of AMD,^6^,^10^ but three previous randomised trials specifically designed to explore this relationship have reported null findings.^11^,^13^ A limitation of observational studies is their inability to account for residual confounding from unmeasured or unknown variables, even after applying advanced statistical methods. This problem becomes more apparent in multifactorial diseases such as AMD, where the level of risk conferred by different genetic, inflammatory, vascular or environmental factors is difficult to quantify.^14^ Random assignment will distribute these characteristics evenly between each treatment group, and thereby minimise the risk of bias.

The ASCEND (A Study of Cardiovascular Events iN Diabetes) trial in a diabetic population^15^ provided the opportunity to undertake randomised comparisons of aspirin and, separately, omega-3 FAs on participant-reported AMD events. Although they share some of the same risk factors, such as age and smoking, diabetes and AMD are not considered to be pathologically related to each other.^16^,^18^ The analyses we describe were part of the ASCEND-Eye substudy.^19^

## Methods

### Trial oversight

Investigators from the Clinical Trial Service Unit at the University of Oxford designed and coordinated ASCEND-Eye. Multi-centre Research Ethics Committee (MREC) approval was granted for the substudy by the North West MREC in October 2016. Members of the writing committee vouch for the faithfulness of the substudy to the data analysis plan.

### Participants and procedures

The design and main results of ASCEND have been described in detail elsewhere.^15 20 21^ Briefly, the trial used mail-based methods to follow-up 15 480 participants recruited in the UK between 2005 and 2011. They comprised men and women with any type of diabetes (excluding gestational diabetes), who were at least 40 years of age, with no previous history of cardiovascular disease, no contraindication to either study treatment and no pre-existing life-limiting medical condition. Using minimised randomisation, eligible participants were assigned in a 2×2 factorial masked design, between 100 mg aspirin daily or a matching placebo and, separately, between 1 g omega-3 fatty acid capsules (containing 460 mg eicosapentaenoic acid (EPA) and 380 mg docosahexaenoic acid (DHA)) daily or a matching placebo. Although not specifically requested on the forms, AMD events could be reported on ASCEND follow-up questionnaires sent to participants every 6 months (see online supplemental material) in response to a general question about ‘any other serious illness or admission to hospital’. Participants could also report AMD events by telephoning the central coordinating office, where a clinician clarified the diagnosis during the call.

While ASCEND was ongoing, ASCEND-Eye sought evidence from participants’ primary care providers to support the adjudication of AMD events by trial clinicians. Events were confirmed against prespecified criteria while masked to the study treatment allocations.^19^ This included documented evidence that an ophthalmologist had confirmed the diagnosis on a date closely matching that given by the participant. Typically, this included clinic letters describing the result of retinal imaging investigations and the subsequent treatment plan. Data on which eye was affected and whether it was the wet or dry form was not routinely collected. Visual Function Questionnaires (VFQ) were sent to all participants who were alive and on web-based or mail-based follow-up when ASCEND ended on 31 July 2017 (see online supplemental material).^19^ The VFQ consisted of two parts: a bespoke first page of questions that explicitly sought new diagnoses of serious eye conditions, including AMD, followed by the standard National Eye Institute Visual Function Questionnaire-25 (NEI-VFQ-25).^22^ The date of the first AMD diagnosis was collected, but information was not requested regarding the phenotypic form or which eye was affected. In total, 8846 out of 11 301 (78.3%) participants who were eligible to receive the VFQ responded.^19^ Events originating from the VFQ were not adjudicated due to the delay between event reporting and the last communication with the participant’s primary care providers. Every randomised participant from ASCEND (n=15 480) was included in the analyses, all of whom gave their written informed consent.

### Outcomes

The effects of allocation to aspirin compared with placebo, and, separately, omega-3 FAs compared with placebo, on time to first confirmed or unrefuted post-randomisation diagnosis of AMD in either eye were a prespecified secondary analysis of the ASCEND-Eye substudy, which has been described in detail previously.^19^ The results of our safety analyses, which included confirmed incidences of sight-threatening eye bleeds in the aspirin randomisation, have also been published elsewhere.^23^

### Statistical analysis

The data analysis plan was publicly available on the trial website (https://ascend.medsci.ox.ac.uk) before unmasking the ASCEND-Eye AMD results.^20^

Age-related macular degeneration is a condition that becomes more common with advancing age. In a meta-analysis of studies that used comparable, standardised protocols and comprised data on nearly 25 000 individuals from high-income countries with predominantly white ethnicity, the estimated age-specific prevalence of AMD was less than 0.5% in those aged between 50 and 60, but this increased to 12% and 16% in men and women over 80 years of age, respectively.^24^ The ASCEND population was relatively young (mean age=63.3; SD 9.2)^15^; therefore, ASCEND-Eye had fewer incident AMD events and thus a lower power to detect a clinically-meaningful treatment effect than would be expected in an older population. The sample size could not be increased because ASCEND-Eye was a substudy of a larger trial. Power calculations^25^ ^26^ based on masked data indicated that there was only 24–41% power to detect 15–20% proportional reductions in AMD events at a two-sided p value of less than 0.05.

The logrank^27 28^ test was used to conduct intention-to-treat analyses of time from randomisation to AMD diagnosis between those randomised to aspirin compared with placebo and, separately, omega-3 FAs versus placebo. Average event rate ratios and their 95% CIs and two-sided p values were calculated using the one-step method from the ‘observed minus expected’ numbers of events (O-E) and their variances (*V*) outputted from the SAS LIFETEST procedure (event rate ratio=exp(O-E/*V*)).^28^ The results are represented graphically in the form of Kaplan-Meier plots. It was anticipated that the factorial design of ASCEND would have little or no effect on the statistical sensitivity with which the effects of each treatment arm could be assessed.^27 29^ Moreover, no clinically significant interactions between the study treatments were anticipated.^30^ The main comparisons of the effect of aspirin were therefore made without stratification by omega-3 FAs allocation (and vice versa for the effect of omega-3 FAs analyses), but post-hoc stratified analyses were carried out to check the validity of this assumption. As AMD was more likely to be recorded on a VFQ form than on an ASCEND follow-up form, analyses were stratified by the availability of a VFQ. No formal adjustments for multiplicity were made for these secondary analyses of ASCEND-Eye.^19^ Therefore, two-tailed p values of less than 0.05 and the 95% CI should be interpreted with caution.

To interpret the impact of adjudication on the AMD outcome, the percentage agreement and Cohen’s Kappa (κ) test of agreement were performed between the pre-adjudicated and post-adjudicated categorisation of AMD events. These prespecified analyses were restricted to the first reports of AMD, where the information source was the participant’s 6 month follow-up questionnaire for ASCEND. Phoned-in events were excluded from the agreement analyses because they may have been more accurately recorded than events reported on in-trial follow-up questionnaires or the VFQ without physician assistance. A post-hoc exploratory analysis assessed the proportion of AMD events derived from the VFQ only, in-trial follow-up questionnaires only, or both.

The Clinical Trial Service Unit at the University of Oxford holds the trial database. All analyses were performed using SAS V. 9.4 and R V. 4.1.2 (R Core Team 2021-11-01) statistical software.

### Patient and public involvement

It was not appropriate or possible to involve patients or the public in the design, or conduct, or reporting, or dissemination plans of our research.

## Results

The baseline characteristics of the ASCEND population and those who returned a VFQ have been described elsewhere.^19^ Key prognostic variables, such as age, smoking status and ethnicity were well-balanced between the randomised groups (table 1 and online supplemental table S1). For the full randomised population of ASCEND, the mean duration of follow-up was 7.4 years (online supplemental table S2), with approximately 114 000 person-years of follow-up split evenly between the treatment arms (online supplemental table S3). Complete morbidity follow-up was available for 15 341 (99.1%) of the randomised population (online supplemental table S4). The study’s average adherence to aspirin and omega-3 FAs was 68.0% and 75.6%, respectively, with similar proportions in the corresponding placebo arms (online supplemental table S5). Adherence declined in the later years of follow-up (online supplemental table S5), with increasing use of non-study aspirin, alternative antiplatelet agents or anticoagulants in the aspirin randomisation (non-study antiplatelet therapy use was 7.9% in the placebo arm and 6.9% in the aspirin arm; 9.6% and 8.4% respectively for non-study antiplatelet or anticoagulant therapy, online supplemental table S6). Study average adherence rates in prespecified baseline characteristic groups were similar in each treatment arm (online supplemental table S7). In both randomisations, ‘Participant wishes’ represented the main reason for discontinuing treatment (online supplemental table S8). There were no significant differences in the number of participants who stopped taking active treatment as compared with the placebo, in either randomisation, overall, or for specific reasons, except that stopping due to minor bleeding or bruising was more prevalent among those who were randomised to aspirin (3.0%) compared with placebo (1.8%).

**Table 1:** Baseline characteristics

Baseline characteristic	Aspirin randomisation				Omega-3 FAs randomisation				Overall (n=15 480)	
	Active (n=7740)		Placebo (n=7740)		Active (n=7740)		Placebo (n=7740)			
Age at randomisation (years)										
Mean (SD)	63.2±9.2		63.3±9.2		63.3±9.2		63.3±9.2		63.3±9.2	
Sex										
Male	4843	(62.6)	4841	(62.5)	4842	(62.6)	4842	(62.6)	9684	(62.6)
Female	2897	(37.4)	2899	(37.5)	2898	(37.4)	2898	(37.4)	5796	(37.4)
Type of diabetes*										
Type 1	458	(5.9)	453	(5.9)	460	(5.9)	451	(5.8)	911	(5.9)
Type 2	7282	(94.1)	7287	(94.1)	7280	(94.1)	7289	(94.2)	14 569	(94.1)
Duration of diabetes (years)										
Median (IQR)	7 (3–13)		7 (3–13)		7 (3–12)		7 (3–13)		7 (3–13)	
Systolic blood pressure (mm Hg)†										
Mean (SD)	136.1±15.2		136.2±15.3		136.2±15.4		136.2±15.1		136.2±15.3	
Diastolic blood pressure (mm Hg)†										
Mean (SD)	77.0±9.4		77.2±9.5		77.1±9.5		77.1±9.5		77.1±9.5	
Body mass index (kg/m^2^)‡										
Mean (SD)	30.8±6.3		30.6±6.3		30.7±6.3		30.8±6.2		30.7±6.3	
Cigarette smoking										
Current	639	(8.3)	640	(8.3)	639	(8.3)	640	(8.3)	1279	(8.3)
Former	3526	(45.6)	3525	(45.5)	3527	(45.6)	3524	(45.5)	7051	(45.5)
Never	3489	(45.1)	3488	(45.1)	3489	(45.1)	3488	(45.1)	6977	(45.1)
Unknown	86	(1.1)	87	(1.1)	85	(1.1)	88	(1.1)	173	(1.1)
Non-study medication										
ACE-inhibitor or ARB	4520	(58.4)	4535	(58.6)	4569	(59.0)	4486	(58.0)	9055	(58.5)
Aspirin use before screening	2740	(35.4)	2768	(35.8)	2744	(35.5)	2764	(35.7)	5508	(35.6)
Thiazide or related diuretic	1480	(19.1)	1477	(19.1)	1448	(18.7)	1509	(19.5)	2957	(19.1)
Calcium channel blocker	1926	(24.9)	1847	(23.9)	1912	(24.7)	1861	(24.0)	3773	(24.4)
Statin	5854	(75.6)	5799	(74.9)	5791	(74.8)	5862	(75.7)	11 653	(75.3)
Total cholesterol (mmol/L)										
Mean (SD)	4.2±0.9		4.2±0.9		4.2±0.9		4.2±0.9		4.2±0.9	
HDL cholesterol (mmol/L)										
Mean (SD)	1.3±0.4		1.3±0.4		1.3±0.4		1.3±0.4		1.3±0.4	
Non-HDL cholesterol (mmol/L)										
Mean (SD)	2.9±0.9		2.9±0.8		2.9±0.9		2.9±0.8		2.9±0.8	
Glycosylated haemoglobin										
IFCC (mmol/mol) mean (SD)	54.7±12.9		54.9±12.9		54.9±13.0		54.7±12.8		54.8±12.9	
CKD-EPI estimated GFR (mL/min/1.73 m^2^)§										
Mean (SD)	85.2±21.1		85.2±21.1		85.2±21.3		85.2±20.8		85.2±21.1	
Urinary albumin:creatinine ratio (mg/mmol)¶										
Median (IQR)	0.56 (0.00–1.33)		0.55 (0.18–1.34)		0.56 (0.18–1.36)		0.55 (0.14–1.32)		0.55 (0.16–1.34)	
Ethnic origin										
White	7467	(96.5%)	7468	(96.5%)	7467	(96.5%)	7468	(96.5%)	14 935	(96.5%)

Figures presented are counts with percentages unless otherwise stated. Percentages may not total 100 because of rounding.

* The presence of type 2 diabetes was based on a broad clinical definition involving the participant’s age at the diagnosis of diabetes, the use of insulin within one1 year after diagnosis, and the body- mass index.

† From blood and urine consent forms, generally before randomizsation.

‡ The body -mass index (the weight in kilograms divided by the square of the height in metres) was based on values for height and weight the participants reported on their randomizsation questionnaires.

§ Calculated from blood cystatin c concentration using the CKD-EPI formula (Inker LA, Schmid CH, Tighiouart H*, et al*. Estimating glomerular filtration rate from serum creatinine and cystatin C. *New England Journal of Medicine* 2012; 367(1): 20–9).

¶ There was an analysis rule in ASCEND which stated that those with a below -detectable threshold albumin component of their urinary albumin creatinine ratio would be recorded as zero. This applied to just over 25% of participants in the active arm of each treatment randomizsation, and to just under 25% of participants in the placebo arm of each treatment randomizsation. Hence the interquartile rangeIQR included zero.

ACE, angiotensin-converting enzyme; ARB, angiotensin receptor blockers; ASCENDA Study of Cardiovascular Events iN DiabetesCKD-EPIChronic Kidney Disease Epidemiology CollaborationFA, fatty acids; GFR, glomerular filtration rate; HDL, high-density lipoprotein; IFCC, International Federation of Clinical Chemistry

The baseline characteristics of VFQ respondents were representative of the full randomised ASCEND population; however, there were statistically significant differences between those included and excluded from the exercise for some characteristics (online supplemental table S9). For example, those excluded were slightly older, and more frequently they were current or former smokers. VFQ forms were completed with a mean of 8.6 years from randomisation (online supplemental table S10). Those who returned a VFQ had better study average adherence, but similar follow-up and patterns of adherence to the overall ASCEND population (online supplemental tables S11–S14).

### Effects of the study treatments on AMD events

AMD was recorded on the VFQ form by 213 out of 8846 (2.4%) participants, but fewer cases were captured by the ASCEND follow-up forms (80/15 480, 0.5%), with 33 AMD events reported by both methods (online supplemental table S15). The proportion of participants who reported an AMD diagnosis on an ASCEND follow-up questionnaire was the same for the VFQ respondents (46/8846, 0.5%) and non-recipient or non-respondents (34/6634, 0.5%, online supplemental table S13). Out of a total of 260 AMD events, 73 were confirmed by adjudication, seven were unrefuted because supporting evidence was not provided by the participants’ General Practitioner (GP) to adjudicate the event, and 180 were unadjudicated, having derived from the VFQ alone.

**Figure 1:**
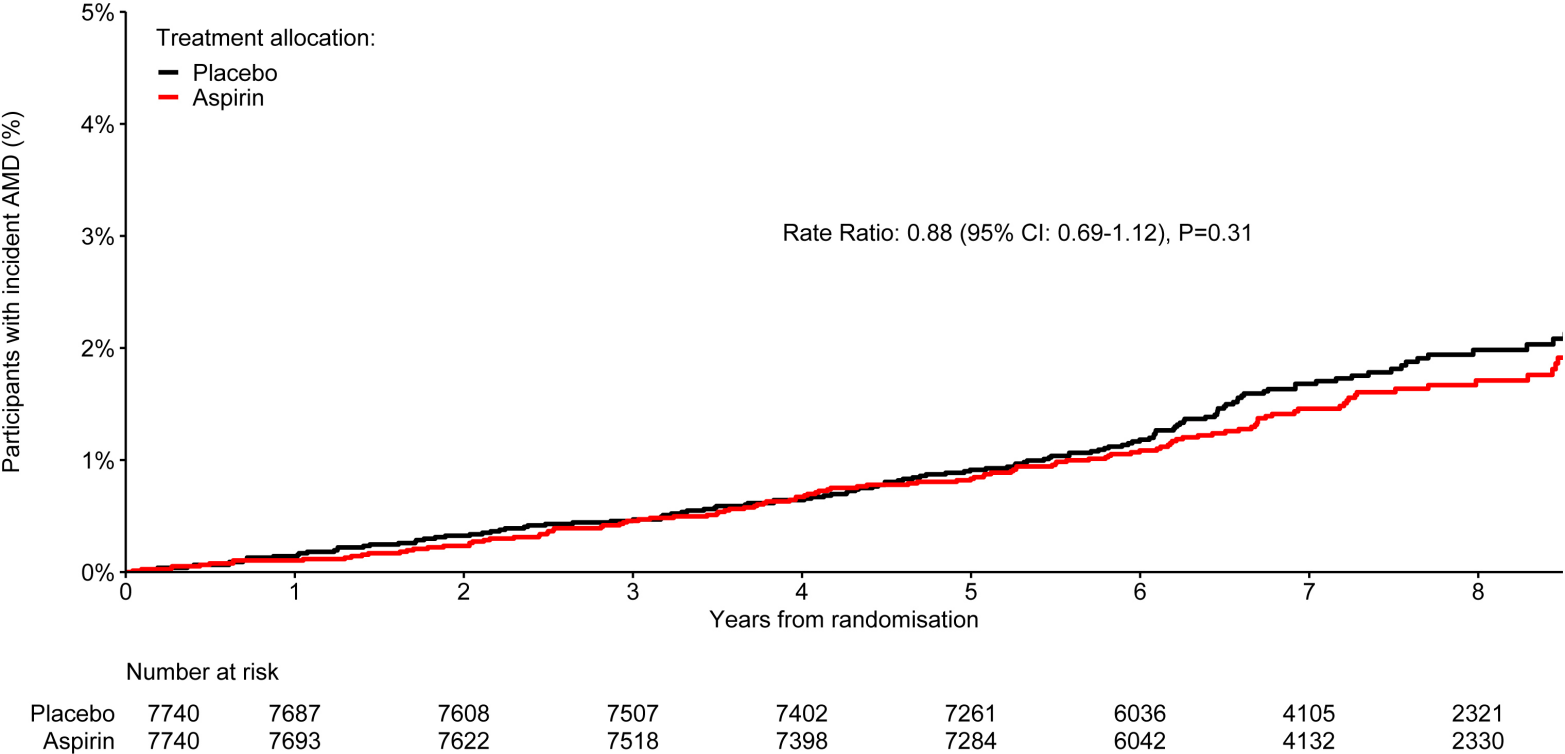
Kaplan-Meier plot of time to confirmed or unrefuted diagnoses of age-related macular degeneration by aspirin allocation. The number of participants at risk at the start of each year of follow-up is shown. The rate ratio is for confirmed or unrefuted AMD diagnoses among participants in the aspirin arm, as compared with those in the placebo arm, stratified by the availability of a Visual Functioning Questionnaire. The corresponding unstratified rate ratio was 0.88 (95% CI 0.69 to 1.13; p=0.31). AMD, age-related macular degeneration.

**Figure 2:**
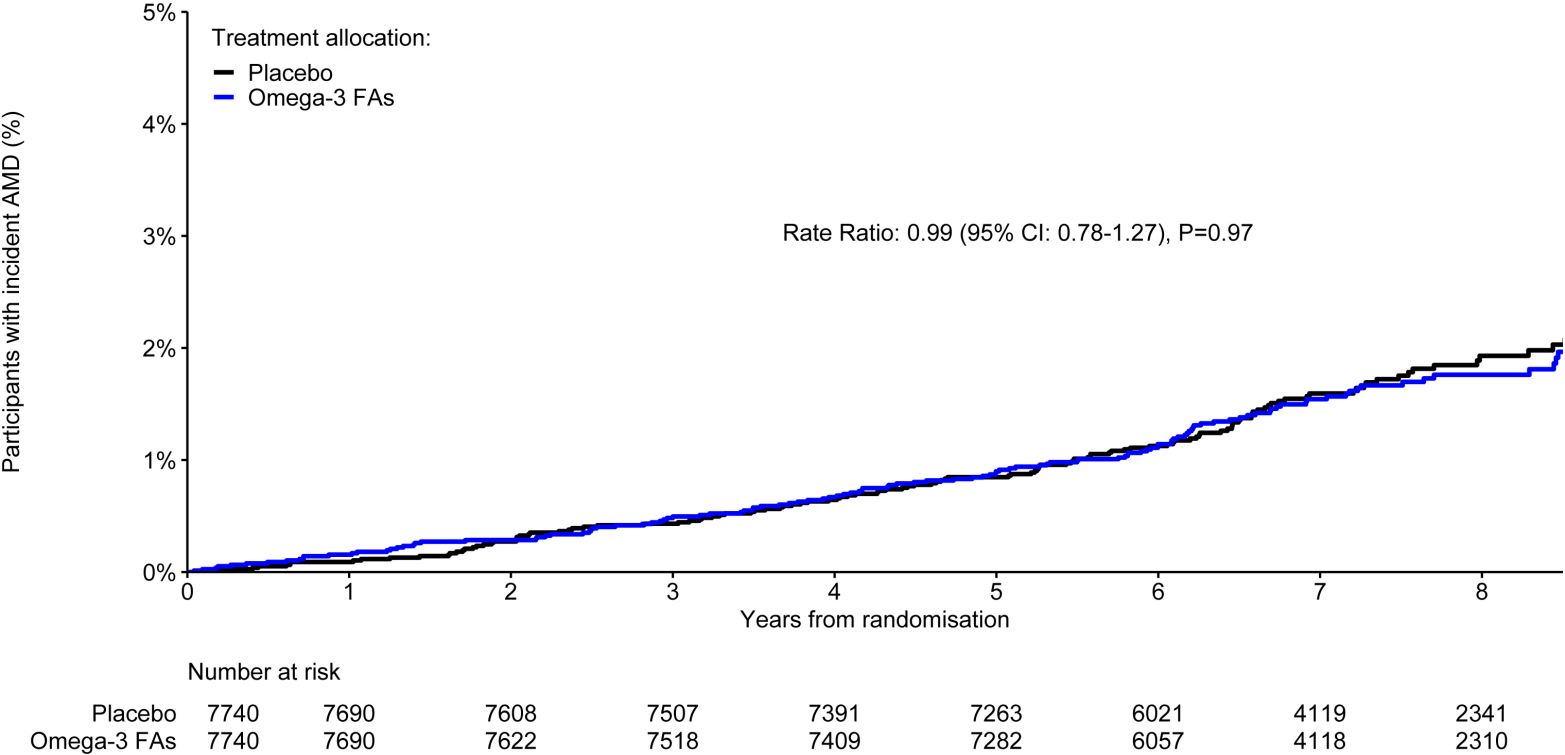
Kaplan-Meier plot of time to confirmed or unrefuted diagnoses of age-related macular degeneration by omega-3 fatty acid allocation. The number of participants at risk at the start of each year of follow-up is shown. The rate ratio is for confirmed or unrefuted AMD diagnoses among participants in the active omega-3 FAs arm, compared with those in the placebo arm, stratified by availability of a Visual Functioning Questionnaire. The corresponding unstratified rate ratio was 1.00 (95% CI 0.78 to 1.27; p=0.99). AMD, age-related macular degeneration; FAs, fatty acids.

There was no significant effect of randomisation to aspirin compared with placebo on AMD diagnoses, which were reported by 122 (1.6%) participants assigned aspirin, compared with 138 (1.8%) assigned placebo (rate ratio 0.88; 95% CI 0.69 to 1.12; p=0.31; figure 1). The survival curves appear to separate after approximately 6 years of follow-up, but the difference did not become statistically significant. Similarly, there was no significant effect of randomisation to omega-3 FAs compared with placebo. AMD occurred in 130 (1.7%) participants allocated to omega-3 FAs, compared with 130 (1.7%) allocated to placebo (rate ratio 0.99; 95% CI 0.78 to 1.27; p=0.97; figure 2). In exploratory analyses, the proportional effects of aspirin and, separately, omega-3 FAs on AMD events did not vary by other treatment assignment (p=0.74 for interaction in the aspirin model and p=0.76 in the omega-3 FA model; online supplemental table S16).

### Effect of adjudication on AMD event categorisation

GPs provided evidence to support the adjudication of 680 out of 725 eye-related adverse events recorded by participants on their ASCEND 6 monthly follow-up forms. The clinical adjudicator agreed with the participant that an AMD event had or had not occurred in 94.9% of cases, with a κ score of 0.71, indicating substantial inter-rater reliability (table 2).^31^

**Table 2:** Effect of adjudication on first participant-reported age-related macular degeneration events

	Adjudicator		
	AMD	No AMD	Total
Participant			
AMD	50	12	62
No AMD	23	595	618
Total	73	607	680

% of agreement=94.9%; Cohen’s κ: 0.71.

Events were restricted to the first reports of AMD recorded on the participant’s six6 monthly follow-up questionnaires for ASCEND and not the VFQ. Phoned-in events and those where supporting evidence was not provided by the participants’ GPGeneral Practitioner to adjudicate the event were excluded.

AMDage-related macular degenerationASCENDA Study of Cardiovascular Events iN DiabetesVFQVisual Function Questionnaire

## Discussion

In the ASCEND-Eye substudy of a large randomised placebo-controlled trial, there were no clinically-meaningful effects of allocation to aspirin 100 mg daily or 1 g omega-3 FAs daily for 7.4 years on confirmed or unrefuted AMD diagnoses.

Aspirin has been considered of potential benefit in AMD owing to its complementary, non-selective and irreversible inhibitive effects on two isoforms of the COX enzyme: COX-1 and COX-2. The long-term suppression of platelet aggregation via acetylation of COX-1 slows atherosclerotic cardiovascular disease progression, which might also protect against retinal arteriolar narrowing and the deposition of lipids in Bruch’s membrane.^32^ Meanwhile, COX-2 inhibition may reduce the platelet-mediated release of vascular endothelial growth factors^33^,^35^ and the expression of pro-inflammatory prostaglandins involved in the biogenesis of drusen.^36 37^ Collectively, these actions might protect against choroidal neovascularisation and disruption of the retinal pigment epithelium. Conversely, the vasoconstrictive effect of COX-2-suppressed prostacyclin synthesis may cause hypoxia in older people with narrowed choroidal blood vessels, becoming the stimulus for neovascularisation and the development of the wet form of AMD.^3^

Our study complements the findings of three other large, double-masked, randomised placebo-controlled trials of aspirin for the primary prevention of cardiovascular events^4 5^ or death, dementia and persistent physical disability.^38^,^40^ The Physician’s Health Study tested 325 mg of aspirin on alternate days in US Physicians aged 40–84 years.^4^ The trial, which stopped early because there were clear reductions in myocardial infarction events, included 22 071 men who did not report having AMD at baseline.^4^ After 5 years of treatment, there were fewer AMD diagnoses in those assigned aspirin compared with those in the placebo group (51 (0.5%) vs 66 (0.6%); rate ratio 0.77; 95% CI 0.54 to 1.11).^4^ Similar results were reported in the Women’s Health Study, which tested 100 mg aspirin on alternate days in female healthcare professionals, aged 45 years or older.^5^ After 10 years of follow-up, among 39 421 women without AMD at baseline, there was a non-significant trend towards a beneficial effect with the active treatment (111 (0.5%) vs 134 (0.7%); HR 0.82; 95% CI 0.64 to 1.06).^5^ Like ASCEND-Eye, in which there is a suggestion that the survival curves may separate in favour of aspirin after 6 years of follow-up, there was a separation of the curves after 3 years of follow-up in the Women’s Health Study, but overall, neither trial reached statistical significance. Finally, a substudy of the ASPirin in Reducing Events in the Elderly (ASPREE) randomised trial tested 100 mg of aspirin daily in 3171 people aged 70 years and older without dementia, independence-limiting physical disability or cardiovascular disease.^41^ AMD status was ascertained by experienced graders using retinal photography and the Beckman classification^42^ of AMD severity. After 3 years of treatment, there were no significant effects of aspirin on cumulative AMD incidence among 1983 participants without AMD at baseline (195 of 1004 (19.4%) vs 187 of 979 (19.1%); relative risk 1.02; 95% CI 0.85 to 1.22).^41^ However, all four trials were underpowered, and the duration of follow-up may have needed to be longer to corroborate observational evidence of harm with more prolonged aspirin exposure (>15 years) and wet AMD.^2 3^ Therefore, obtaining further data regarding the effects of aspirin on AMD over a more extended follow-up period may be important. It may be possible to obtain long-term follow-up data of our participants by linking to their electronic UK National Ophthalmology Database AMD Audit records if an application to secure Section 251 exemption to access patient identifiers is approved.^43^ If it was confirmed that harmful effects take longer to emerge, it might be appropriate to offer older people prescribed aspirin more frequent eye examinations, given the potential to prevent rapid visual loss from wet AMD with anti-Vascular Endothelial Growth Factor (VEGF) therapies.

Experimental studies have shown that omega-3 FAs, in particular DHA, could have a protective role against the development or progression of AMD via complex antiangiogenic and antioxidant pathways, localised to the retina.^44^ In support of this, most, but not all, observational studies have found that affected individuals tend to recall a lower consumption of fish or omega-3 FAs.^7 9 45 46^ However, our results are consistent with three previous randomised trials of omega-3 FAs, which also failed to demonstrate any effects.^11^,^13^ The Age-Related Eye Disease Study-2 (AREDS2) trial randomised 4203 men and women aged 50–85 with bilateral early disease or late AMD in only one eye, in a 2×2 factorial design between 10 mg lutein plus 2 mg zeaxanthin daily or a matching placebo and, separately, between omega-3 FA capsules (containing 650 mg EPA and 350 mg DHA) daily or a matching placebo.^11^ The primary outcome was incident wet or dry AMD identified using annual mydriatic fundus photographs.^11^ During 4.9 years of follow-up, allocation to active omega-3 FAs did not prevent AMD progression: 979 out of 3491 eyes (28.0%) in the active treatment group developed late disease, compared with 961 out of 3400 eyes (28.3%) in the placebo group (HR 0.98; 95% CI 0.89 to 1.08; p=0.74).^11^ Similar results were reported in the VITamin D and omega-3 triAL (VITAL-AMD) study, which randomised 25 871 men over 50 and women over 55 years of age, in a 2×2 factorial masked design, to 2000 units of cholecalciferol daily or a matching placebo and, separately, to an omega-3 FA supplement (containing 460 mg EPA and 380 mg DHA) daily or a matching placebo.^13^ The primary outcome of total AMD was a composite of incident AMD or AMD progression based on participant-reported events confirmed by medical record review.^13^ Over 5.3 years of follow-up, allocation to active omega-3 FAs did not reduce the total number of participants who developed AMD compared with the placebo: 157 (1.2%) versus 167 (1.3%), respectively; HR 0.93; 95% CI 0.73 to 1.17.^13^ Finally, the Nutritional AMD Treatment-2 (NAT-2) study compared the efficacy of DHA-enriched supplementation for the prevention of wet AMD.^12^ In total, 263 men and women aged 55–85 years with bilateral drusen or wet AMD in just one eye were randomised to a daily omega-3 FA supplement (containing 270 mg EPA and 840 mg DHA) or matching placebo.^12^ Participants underwent baseline and annual fluorescein angiography examinations over a 3-year follow-up period.^12^ There were no beneficial effects of omega-3 FAs on the primary efficacy outcome of time to the first occurrence of wet AMD: 19.5±10.9 months in the active group versus 18.7±10.6 months in the placebo group (HR 0.89; 95% CI 0.55 to 1.42).^12^ However, only a per-protocol analysis was performed instead of intention-to-treat, which invalidates the underlying randomisation and may be prone to bias.

ASCEND-Eye has several strengths that facilitated a robust assessment of the effects of the study treatments on AMD, including its randomised design, well-defined exposure, a large number of participants and long duration of near-complete follow-up. Our study also has some limitations. Although the study average adherence was relatively good, poor adherence in the later years of follow-up, along with drop-ins to treatment in the placebo arm of the aspirin randomisation by those prescribed non-study antiplatelet therapy, may have shifted risk estimates towards the null. Like the earlier studies, ASCEND-Eye was underpowered to detect modest but plausible effects of each treatment. The lack of power and lack of overall effect meant it was not appropriate to consider the effects of aspirin or omega-3 FAs in subgroups of important prognostic risk factors, such as age, gender or smoking.

AMD diagnoses that were eligible to be adjudicated were confirmed by medical record review, but confirmation by retinal photography remains the gold standard for detailed phenotyping of early and late disease, and wet and dry AMD subtypes. Since the AMD status of participants at baseline was unknown, we could not ascertain their disease progression or assess any possible treatment effects on disease progression. However, it seems unlikely that a treatment may have a large effect on progression and no effect on incidence.

The adjudication process had little effect on the categorisation of AMD events, which supported the decision not to adjudicate events derived from the VFQ. However, there was a difference in the psychometric performance of the in-trial and VFQ questionnaires. While the proportion of AMD events recorded on in-trial questionnaires was the same among those who did and did not receive the VFQ, the VFQ captured substantially more events than the in-trial questionnaires. This probably reflects the lack of any explicit questions about AMD on the ASCEND follow-up questionnaire. Therefore, the possibility of some under-reporting of events by participants who were ineligible to receive or did not respond to the VFQ cannot be excluded. These differences would further reduce the study’s power, but they would not be expected to bias the results in favour of a particular treatment.

Next, although our findings are internally valid, their generalisability in a real-world setting may be limited by a lack of ethnic diversity in the ASCEND study population. Finally, although diabetes and AMD are not considered to be pathologically related to each other,^17^ the possibility cannot be excluded that the pharmacodynamic effects of each study treatment differ in those with diabetes compared with those without it.

In conclusion, we found no clinically-meaningful effects of taking aspirin or omega-3 FAs for the prevention of AMD. Our study overcomes some of the methodological limitations of previous observational studies, providing randomised evidence that could contribute to future meta-analyses.

## supplementary material

10.1136/bmjopen-2024-090605 online supplemental table 1

## Data Availability

Data are available upon reasonable request.
