# Supplementary material for: Effects of aspirin and omega-3 fatty acids on age-related macular degeneration in ASCEND-Eye: a randomised placebo-controlled trial in a population with diabetes
Source: BMJ Open. 2025 Feb 26;15(2):e090605. doi: 10.1136/bmjopen-2024-090605 (PMC11865773; doi:10.1136/bmjopen-2024-090605)
Supplement: online supplemental table 1 [file bmjopen-15-2-s001.pdf]

Supplementary Appendix

This supplement contains the following items:

Contents

ASCEND-Eye Collaborative Group........ 2
Table S1 Baseline Characteristics of those VFQ Responders and Non-Responders by Eligibility to be sent the VFQ........ 4
Table S2 Duration of Follow-Up- All Randomized ASCEND Participants........ 7
Table S3 Person-Years of Follow-up, Overall and by Follow-Up Method – All Randomized ASCEND Participants........ 7
Table S4 Completeness of Follow-Up – All Randomized ASCEND Participants........ 7
Table S5 Reported Definite or Probable Adherence with Study Treatment Stratified by Years Post-Randomization – All Randomized ASCEND Participants........ 8
Table S6 Use of Anti-platelet or Anti-coagulant Therapy in the Aspirin Randomization during Follow-Up – All Randomized ASCEND Participants........ 8
Table S7 Study Average Reported Definite or Probable Adherence in each Treatment Arm, Overall and by Baseline Characteristics – All Randomized ASCEND Participants........ 9
Table S8 Reasons for Stopping Treatment – All Randomized ASCEND Participants........ 12
Table S9 Heterogeneity of Baseline Characteristics between VFQ Responders and Non-Responders of among those who were Eligible to be sent the VFQ (n=11,301)........ 13
Table S10 Duration Follow-up in the Visual Function Questionnaire Respondents........ 16
Table S11 Reported Definite or Probable Adherence with Study Treatment Stratified by Years Post-Randomization – Visual Function Questionnaire Respondents........ 17
Table S12 Use of Anti-platelet or Anti-coagulant Therapy in the Aspirin Arm during Follow-Up – Visual Function Questionnaire Respondents........ 17
Table S13 Study Average Reported Definite or Probable Adherence in each Treatment Arm, Overall and by Baseline Characteristics – Visual Function Questionnaire Respondents........ 18
Table S14 Reasons for Stopping Treatment – Visual Function Questionnaire Respondents........ 21
Table S15 Source of Age-Related Macular Degeneration Events........ 22
Table S16 Effects of Aspirin and Omega-3 Fatty Acids on Age-Related Macular Degeneration Events Stratified by the Other Treatment in the Factorial Design........ 22
ASCEND Follow-Up Questionnaire........ 23
ASCEND-Eye Visual Function Questionnaire........ 24

ASCEND-Eye Collaborative Group

Writing Committee

Emily Sammons (*Corresponding author)*, Louise Bowman, William Stevens, Georgina Buck, Imen Hammami, Sarah Parish, Jane Armitage

Steering Committee

Chairman: R Collins, *Study coordinator:* J Armitage; *Clinical coordinator:* L Bowman; *Statisticians:* S Parish, R Peto; *Administrative coordinator:* J Barton; *Lay member:* D Simpson; *Other members:* A Adler, T Aung, C Baigent, HJ Bodansky, A Farmer, R Haynes, R McPherson, M Mafham, HAW Neil, N Samani, P Sleight, P Weissberg.

Data Monitoring Committee

Chair: P Sandercock, *Members:* H Gerstein, R Gray, C Hennekens.

Coordinating Office (Clinical Trial Service Unit, Nuffield Department of Population Health, University of Oxford):

Administration and support: J Barton, L Fletcher, K Murphy (coordinators); S Hurley, R Lee, S Pickworth, M Willett, M Wincott.

Clinical support and adjudication: J Armitage, L Bowman, M Mafham, E Sammons

Statistics and computing: M Lay, S Parish; G Buck, A Murawska, W Stevens, K Wallendszus, A Young, I Hammami

Research support: K Melham, G Brown, J Latham-Mollart, A Brewer

Collaborators

Ophthalmologists: P Scanlon[1], P Patel[2]

Public Health England (now the UK Health Security Agency): M Olson[3]

Public Health Wales[4]: J Kay, S Banerjee, L Evans, A Davies, M Griffiths, H Clayton

Health Intelligence Ltd[5]: P Kirby. M Pennington[8], D Clarke

Northgate Ltd[6]: J Anslow, A Hallam, J Witts, S Egan, A Wharton

Cheshire DESP[7]: A Sachdev, A Derbyshire, E Williamson, K Hepplestone

East and North Hertfordshire DESP[8]: S Mithra, S Oliver, P Wiatrak-Olszewska

Greater Nottingham DESP[9]: T Gazis, K Alvey, E Wu

Humber DESP[10]: H Cook, N Gregory, P Parkinson

North East London DESP[11]: J Anderson, L Bolter

North Nottinghamshire DESP[12]: P Maharajan, R McFee, L Allsop, D Sowter, D Hodgson

North Yorkshire DESP[13]: J Thow, J Featonby, R Furnival

Oxfordshire DESP[14]: P Scanlon, H Lipinski, H Benjamin, T McAfee

South East Sussex DESP[15]: E Payne, L Still

Funding

The Macular Society[16]
British Heart Foundation[17]

1. Department of Ophthalmology, Gloucestershire Hospitals NHS Foundation Trust, Cheltenham General Hospital, Sandford Road, Cheltenham, GL53 7AN
2. Moorfields Eye Hospital, 162 City Road, London, EC1V 2PD
3. Health Information Consulting Ltd, Low Barn, Homestall Lane, Faversham, Kent, ME13 8UT
4. Public Health Wales Research and Evaluation Division, Knowledge Directorate, Floor 5, 2 Capital Quarter, Tyndall Street, Cardiff, CF10 4BZ
5. InHealth Intelligence, Unity House, Road 5, Winsford Industrial Estate, Winsford, Cheshire, CW7 3RB
6. Northgate Public Services (UK) Ltd, Crome Lea Business park, Madingley road, Coton, CB23 7PH
7. Cheshire Diabetic Eye Screening Programme, Eagle Bridge Health and Wellbeing Centre, Dunwoody Way, Crewe, CW1 3AW
8. East and North Hertfordshire Diabetic Eye Screening Programme, Administration Centre H4, Hertford County Hospital, North Road, Hertford, SG14 1LP
9. Greater Nottinghamshire Diabetic Eye Screening Programme, 2nd Floor, Ropewalk House, 113 The Ropewalk, Nottingham, NG1 5DU
10. Humber Diabetic Eye Screening Programme, Alderson House, Hull Royal Infirmary, Hull, HU3 2JZ
11. North East London Diabetic Eye Screening Programme, The Eye Screening Centre, Homerton University Hospital, Homerton Row, London
12. North Nottinghamshire Diabetic Eye Screening Programme, Kings Mill Hospital, Trust Admin Building - Level 2, Mansfield Road, Sutton In Ashfield, Notts, NG17 4JL
13. North Yorkshire Diabetic Eye Screening Programme, 2 Cayley Court, George Cayley Drive, Clifton Moor, York, YO30 4WH
14. Oxfordshire Diabetic Eye Screening Programme, Level 0, West Wing, John Radcliffe Hospital, Headley Way, Headington, Oxford, OX3 9DU
15. South East Sussex Diabetic Eye Screening Programme, Bexhill Hospital, East Sussex Healthcare NHS Trust, Hollier's Hill, Bexhill-on-Sea, TN40 2DZ
16. Macular Society, Crown Chambers, South Street, Andover SP10 2BN
17. British Heart Foundation, Greater London House, 5th Floor, 180 Hampstead Road, London, UK, NW1 7AW

Table S1 Baseline Characteristics of those VFQ Responders and Non-Responders by Eligibility to be sent the VFQ

	Eligible to be sent the VFQ (n=11,301)		Ineligible to be sent the VFQ (n=4179)
**Baseline Characteristic**	**Responders (n=8846)***	**Non-responders (n=2455)**	
**Age at randomisation (years)**			
Mean (SD)	62.5±8.3	62.8±9.0	65.2±10.7
<60	3339 (37.7%)	901 (36.7%)	1350 (32.3%)
≥60<70	3860 (43.6%)	1024 (41.7%)	1363 (32.6%)
≥70	1647 (18.6%)	530 (21.6%)	1466 (35.1%)
**Sex**			
Male	5529 (62.5%)	1550 (63.1%)	2605 (62.3%)
Female	3317 (37.5%)	905 (36.9%)	1574 (37.7%)
**Type of diabetes**			
Type 1	559 (6.3%)	138 (5.6%)	214 (5.1%)
Type 2	8287 (93.7%)	2317 (94.4%)	3965 (94.9%)
**Duration of diabetes (years)**			
Median (IQR)	7(3-12)	7 (4-13)	7 (4-13)
≥0<5 years	2918 (33.0%)	751 (30.6%)	1222 (29.2%)
≥5<10 years	2544 (28.8%)	690 (28.1%)	1100 (26.3%)
≥10<20 years	1952 (22.1%)	552 (22.5%)	1033 (24.7%)
≥20 years	1020 (11.5%)	313 (12.7%)	529 (12.7%)
Unknown	412 (4.7%)	149 (6.1%)	295 (7.1%)
**Diabetes management**			
Diet only	1508 (17.0%)	396 (16.1%)	625 (15.0%)
Oral hypoglycaemic agent(s) only	5166 (58.4%)	1416 (57.7%)	2438 (58.3%)
Insulin +/- oral hypoglycaemic agent(s)	2172 (24.6%)	643 (26.2%)	1116 (26.7%)
**Participant-reported diabetic retinopathy**			
Yes	1628 (18.4%)	537 (21.9%)	858 (20.5%)
No	7151 (80.8%)	1895 (77.2%)	3267 (78.2%)
Unknown	67 (0.8%)	23 (0.9%)	54 (1.3%)
**Participant-reported treatment for hypertension**			
Yes	5386 (60.9%)	1534 (62.5%)	2613 (62.5%)
No	3405 (38.5%)	904 (36.8%)	1526 (36.5%)
Unknown	55 (0.6%)	17 (0.7%)	40 (1.0%)
**Systolic blood pressure (mmHg)**			
Mean (SD)	135.8±14.9	136.3±15.2	136.8±15.9
<130	2023 (22.9%)	522 (21.3%)	849 (20.3%)
≥130<140	1822 (20.6%)	491 (20.0%)	778 (18.6%)
≥140	2626 (29.7%)	720 (29.3%)	1209 (28.9%)
Unknown	2375 (26.8%)	722 (29.4%)	1343 (32.1%)
**Diastolic blood pressure (mmHg)**			
Mean (SD)	77.4±9.2	77.0±9.6	76.4±9.9
<75	2371 (26.8%)	650 (26.5%)	1202 (28.8%)
≥75 <85	2788 (31.5%)	753 (30.7%)	1078 (25.8%)
≥85	1311 (14.8%)	326 (13.3%)	554 (13.3%)
Unknown	2376 (26.9%)	726 (29.6%)	1345 (32.2%)
**Body mass index (kg/m^2)**			
Mean (SD)	30.6±6.1	30.9±6.3	30.9±6.5
<25	1313 (14.8%)	309 (12.6%)	627 (15.0%)
≥25, <30	3230 (36.5%)	901 (36.7%)	1398 (33.5%)
≥30<35	2429 (27.5%)	688 (28.0%)	1123 (26.9%)
≥35	1619 (18.3%)	472 (19.2%)	870 (20.8%)
Unknown	255 (2.9%)	85 (3.5%)	161 (3.9%)
**Cigarette smoking**			
Current	555 (6.3%)	224 (9.1%)	500 (12.0%)
Former	3947 (44.6%)	1133 (46.2%)	1971 (47.2%)
Never	4243 (48.0%)	1067 (43.5%)	1667 (39.9%)
Unknown	101 (1.1%)	31 (1.3%)	41 (1.0%)

Baseline Characteristic	Eligible to be sent the VFQ (n=11,301) Responders (n=8846)*	Eligible to be sent the VFQ (n=11,301) Non-responders (n=2455)	Ineligible to be sent the VFQ (n=4179)
**Non-study medication**			
ACE-inhibitor or ARB	5115 (57.8%)	1461 (59.5%)	2479 (59.3%)
Aspirin use before screening	3157 (35.7%)	879 (35.8%)	1472 (35.2%)
Thiazide or related diuretic	1639 (18.5%)	465 (18.9%)	853 (20.4%)
Calcium channel blocker	2088 (23.6%)	605 (24.6%)	1080 (25.8%)
Statin	6769 (76.5%)	1862 (75.8%)	3022 (72.3%)
**Total cholesterol (mmol/L)**			
Mean (SD)	4.1±0.9	4.1±0.9	4.2±0.9
<4	2681 (30.3%)	704 (28.7%)	1169 (28.0%)
≥4 <5	2246 (25.4%)	595 (24.2%)	954 (22.8%)
≥5	817 (9.2%)	226 (9.2%)	427 (10.2%)
Not available	3102 (35.1%)	930 (37.9%)	1629 (39.0%)
**HDL cholesterol (mmol/L)**			
Mean (SD)	1.3±0.4	1.3±0.4	1.2±0.4
<1	1222 (13.8%)	343 (14.0%)	605 (14.5%)
≥1<1.5	3202 (36.2%)	870 (35.4%)	1451 (34.7%)
≥1.5	1310 (14.8%)	307 (12.5%)	490 (11.7%)
Not available	3112 (35.2%)	935 (38.1%)	1633 (39.1%)
**Non-HDL cholesterol (mmol/L)**			
Mean (SD)	2.9±0.8	2.9±0.8	3.0±0.9
<2.5	2053 (23.2%)	505 (20.6%)	832 (19.0%)
≥2.5 <3.5	2558 (28.9%)	716 (29.2%)	1119 (26.8%)
≥3.5	1123 (12.7%)	299 (12.2%)	595 (14.2%)
Not available	3112 (35.2%)	935 (38.1%)	1633 (39.1%)
**Glycosylated haemoglobin - HbA1c**			
IFCC (mmol/mol) mean (SD)	54.0±12.2	55.6±13.5	56.1±14.0
DCCT (%) mean (SD)	7.1±1.1	7.2±1.2	7.3±1.3
<48 (6.5)	1957 (22.1%)	496 (20.2%)	819 (19.6%)
≥48 (6.5), <64 (8.0)	2792 (31.6%)	697 (28.4%)	1175 (28.1%)
≥64 (8.0)	990 (11.2%)	332 (13.5%)	555 (13.2%)
Not available	3107 (35.1%)	930 (37.9%)	1630 (39.0%)
**CKD-EPI estimated GFR (ml/min/1.73m^2)**			
Mean (SD)	87.8±19.8	84.8±20.4	79.5±23.1
≥90	2902 (32.8%)	671 (27.3%)	950 (22.7%)
≥60<90	2299 (26.0%)	659 (26.8%)	1058 (25.3%)
<60	540 (6.1%)	194 (7.9%)	542 (13.0%)
Not available	3105 (35.1%)	931 (37.9%)	1629 (39.0%)
**Urinary albumin:creatinine ratio† (mg/mmol)**			
Median (IQR)	0.50 (0.00-1.14)	0.59 (0.16-1.40)	0.71 (0.27-1.88)
<3	5120 (57.9%)	1313 (53.5%)	2093 (50.1%)
≥3	613 (6.9%)	197 (8.0%)	438 (10.5%)
Not available	3113 (35.2%)	945 (38.5%)	1648 (39.4%)
**Townsend Deprivation Index**			
<-3	3101 (35.1%)	764 (31.1%)	1239 (29.7%)
≥-3<0	3588 (40.6%)	930 (37.9%)	1504 (36.0%)
≥0<2	1071 (12.1%)	364 (14.8%)	602 (14.4%)
≥2<4	625 (7.1%)	244 (9.9%)	446 (10.7%)
≥4<6	323 (3.7%)	112 (4.6%)	268 (6.4%)
≥6	118 (1.3%)	37 (1.5%)	106 (2.5%)
Unknown	20 (0.2%)	4 (0.2%)	14 (0.9%)
**Ethnic origin**			
White	8564 (96.8%)	2346 (95.6%)	4025 (96.3%)
Indian/Pakistani/Bangladeshi	107 (1.2%)	39 (1.6%)	38 (0.9%)
African/Caribbean	54 (0.6%)	29 (1.2%)	57 (1.4%)
Other/unknown	121 (1.4%)	41 (1.7%)	59 (1.4%)

ACE = angiotensin-converting enzyme; ARB = angiotensin receptor blockers; DCCT = Diabetes Control and Complications Trial; FAs = Fatty acids; GFR = Glomerular Filtration Rate; HDL=High-density lipoprotein; IFCC =International Federation of Clinical Chemistry; IQR = Interquartile range; SD = Standard Deviation

*Out of 8846 VFQ respondents, 7 answered a bespoke first page of questions that sought incident eye diagnoses but did not complete the NEI-VFQ-25, and 8839 completed both parts of the form. Separate analyses of the baseline characteristics for the 8839 NEI-VFQ-25 respondents have not been performed because it is unlikely that excluding those 7 participants would make meaningful differences to the average results.

†There was an analysis rule in ASCEND which stated that those with a below detectable threshold albumin component of their urinary albumin:creatinine ratio, would be recorded as zero. This applied to just over 25% of participants with no baseline eye screening records.

Categories that were “unknown” or “not available” were excluded from balance diagnostic testing.

Percentages may not total 100 because of rounding.

Table S2 Duration of Follow-Up- All Randomized ASCEND Participants

	Aspirin Randomization		Omega-3 FAs Randomization		
	**Active (n=7740)**	**Placebo (n=7740)**	**Active (n=7740)**	**Placebo (n=7740)**	**Overall (n=15,480)**
**Mean(SD) length of follow-up (years)**	7.4 (1.8)	7.4 (1.8)	7.4 (1.8)	7.4 (1.8)	7.4 (1.8)
**Median (IQR) length of follow-up (years)**	7.3 (6.4-8.5)	7.3 (6.4-8.5)	7.3 (6.4-8.5)	7.3 (6.4-8.5)	7.3 (6.4-8.5)

FAs=Fatty Acids; IQR=Interquartile range; SD=Standard deviation.

Table S3 Person-Years of Follow-up, Overall and by Follow-Up Method – All Randomized ASCEND Participants

	Aspirin Randomization		Omega-3 FAs Randomization		
	**Active (n=7740)**	**Placebo (n=7740)**	**Active (n=7740)**	**Placebo (n=7740)**	**Overall (n=15,480)**
**Follow-up method**					
Participant	43,627.0 (76.5%)	43,053.7 (75.6%)	43,416.6 (76.1%)	43,264.1 (76.0%)	86,680.7 (76.1%)
General practitioner	8018.3 (14.1%)	8423.6 (14.8%)	8274.2 (14.5%)	8167.7 (14.3%)	16,441.9 (14.4%)
Record linkage	1742.2 (3.1%)	1674.1 (2.9%)	1685.9 (3.0%)	1730.4 (3.0%)	3416.3 (3.0%)
Died with complete follow-up	3309.2 (5.8%)	3410.3 (6.0%)	3286.2 (5.8%)	3433.3 (6.0%)	6719.5 (5.9%)
Incomplete follow-up information	303.7 (0.5%)	383.4 (0.7%)	358.7 (0.6%)	328.4 (0.6%)	687.1 (0.6%)
**Total**	**57,000.4**	**56,945.0**	**57,021.5**	**56,923.9**	**113,945.4**

FAs=Fatty Acids. All figures represent person-years of follow-up. Percentages may not total 100 because of rounding.

Table S4 Completeness of Follow-Up – All Randomized ASCEND Participants

	Aspirin Randomization		Omega-3 FAs Randomization		
**Final follow-up status**	**Active n=7740**	**Placebo n=7740**	**Active n=7740**	**Placebo n=7740**	**Overall n=15,480**
**Complete follow-up information**	**7671 (99.1%)**	**7670 (99.1%)**	**7672 (99.1%)**	**7669 (99.1%)**	**15,341 (99.1%)**
Final FU completed by participant or carer	5763 (74.5%)	5682 (73.4%)	5730 (74.0%)	5715 (73.8%)	11,445 (73.9%)
Final FU completed by GP	999 (12.9%)	1045 (13.5%)	1032 (13.3%)	1012 (13.1%)	2044 (13.2%)
Final FU completed by registry linkage	224 (2.9%)	218 (2.8%)	221 (2.9%)	221 (2.9%)	442 (2.9%)
Died (FU for morbidity complete)	685 (8.9%)	725 (9.4%)	689 (8.9%)	721 (9.3%)	1410 (9.1%)
**Incomplete follow-up information**	**69 (0.9%)**	**70 (0.9%)**	**68 (0.9%)**	**71 (0.9%)**	**139 (0.9%)**
Consent withdrawn	35 (0.5%)	30 (0.4%)	26 (0.3%)	39 (0.5%)	65 (0.4%)
Moved abroad	17 (0.2%)	14 (0.2%)	19 (0.2%)	12 (0.2%)	31 (0.2%)
Died (FU for morbidity not complete)	9 (0.1%)	13 (0.2%)	11 (0.1%)	11 (0.1%)	22 (0.1%)
No final FU information	8 (0.1%)	13 (0.2%)	12 (0.2%)	9 (0.1%)	21 (0.1%)

FAs=Fatty Acids; FU=Follow up; GP=General practitioner. Percentages represent the proportion each follow-up method contributed to the total number of person-years of follow-up. Percentages may not total 100 because of rounding.

Table S5 Reported Definite or Probable Adherence with Study Treatment Stratified by Years Post-Randomization – All Randomized ASCEND Participants

	Aspirin Randomization			Omega-3 FAs Randomization		
	**Active (%)**	**Placebo (%)**	**Overall (%)**	**Active (%)**	**Placebo (%)**	**Overall (%)**
**Years post-randomization**						
<3	82.0%	81.7%	81.9%	86.1%	85.7%	85.9%
≥3<5	66.0%	64.9%	65.5%	74.1%	73.6%	73.9%
≥5<7	56.2%	55.1%	55.6%	66.9%	66.2%	66.6%
≥7	46.2%	45.6%	46.0%	58.9%	58.1%	58.5%
**All**	**68.0%**	**67.3%**	**67.7%**	**75.6%**	**75.1%**	**75.4%**

FAs=Fatty Acids.
Criteria used to define definite or probable adherence:

- Definitely adherent = participant reported taking treatment every or most days during the follow-up period.
- Probably adherent = participant was previously adherent, is still receiving medication and has not reported stopping treatment within the last seven months.

Table S6 Use of Anti-platelet or Anti-coagulant Therapy in the Aspirin Randomization during Follow-Up – All Randomized ASCEND Participants

	Aspirin Randomization				
	**Active (n=7740)**			**Placebo (n=7740)**	
	**Study aspirin**	**Study aspirin or non-study anti-platelet**	**Study aspirin or non-study anti-platelet agent or anti-coagulant**	**Non-study anti-platelet**	**Non-study anti-platelet agent or anti-coagulant**
**Years post-randomization**					
<3	82.0%	85.3%	85.8%	4.1%	4.6%
≥3<5	66.0%	73.4%	74.9%	8.8%	10.5%
≥5<7	56.2%	65.9%	68.3%	11.0%	13.9%
≥7	46.2%	59.1%	61.9%	12.9%	16.3%
**All**	**68.0%**	**74.9%**	**76.4%**	**7.9%**	**9.6%**

Table S7 Study Average Reported Definite or Probable Adherence in each Treatment Arm, Overall and by Baseline Characteristics – All Randomized ASCEND Participants

Baseline Characteristic	Aspirin Randomization			Omega-3 FAs Randomization		
	Active (n =7740)	Placebo (n=7740)	Overall (n=15,480)	Active (n=7740)	Placebo (n=7740)	Overall (n=15,480)
**Age at randomization (years)**						
<60	69.3%	68.5%	68.9%	74.9%	74.7%	74.8%
≥60<70	69.3%	68.9%	69.1%	77.3%	77.4%	77.4%
≥70	63.5%	62.3%	62.9%	73.6%	71.7%	72.6%
**Sex**						
Male	69.8%	68.9%	69.3%	77.8%	77.2%	77.5%
Female	65.2%	64.7%	64.9%	72.0%	71.6%	71.8%
**Type of diabetes***						
Type 1	68.7%	69.9%	69.3%	77.3%	74.5%	75.9%
Type 2	68.0%	67.1%	67.6%	75.5%	75.1%	75.3%
**Duration of diabetes (years)**						
≥0<5 years	69.7%	68.6%	69.2%	76.4%	76.5%	76.4%
≥5<10 years	68.2%	66.6%	67.4%	75.8%	74.3%	75.1%
≥10<20 years	67.2%	67.7%	67.5%	75.6%	75.3%	75.4%
≥20 years	66.9%	68.1%	67.5%	76.7%	74.9%	75.8%
Unknown	63.4%	60.2%	61.8%	68.0%	70.6%	69.3%
**Diabetes management**						
Diet only	67.1%	65.6%	66.3%	75.9%	73.2%	74.5%
Oral hypoglycaemic agent(s) only	68.4%	67.7%	68.0%	75.6%	75.9%	75.8%
Insulin +/- oral hypoglycaemic agent(s)	67.9%	67.5%	67.7%	75.6%	74.4%	75.0%
**Participant-reported diabetic retinopathy**						
Yes	66.4%	65.9%	66.2%	75.6%	74.9%	75.3%
No	68.5%	67.7%	68.1%	75.6%	75.1%	75.4%
**Participant-reported treatment for hypertension**						
Yes	67.6%	67.2%	67.4%	76.2%	74.8%	75.5%
No	68.8%	67.5%	68.2%	74.7%	75.5%	75.1%
**Systolic blood pressure (mmHg) †**						
<130	69.8%	70.1%	69.9%	76.9%	76.9%	76.9%
≥130<140	69.9%	69.1%	69.6%	78.1%	76.8%	77.5%
≥140	68.5%	68.3%	68.4%	77.2%	77.4%	77.3%
Unknown	64.9%	62.9%	63.9%	71.4%	70.2%	70.8%
**Diastolic blood pressure (mmHg) †**						
<75	79.9%	80.3%	80.1%	88.2%	87.7%	87.9%
≥75 <85	70.6%	69.9%	70.3%	78.1%	78.0%	78.1%
≥85	68.4%	69.4%	68.9%	76.6%	77.8%	77.2%
Unknown	65.0%	62.9%	63.9%	71.4%	70.2%	70.8%
**Body mass index (kg/m^2) ‡**						
<25	68.6%	69.2%	68.9%	77.1%	76.0%	76.6%
≥25, <30	68.6%	68.3%	68.5%	76.2%	76.5%	76.3%
≥30<35	68.2%	66.5%	67.3%	74.9%	74.9%	74.9%
≥35	66.9%	65.7%	66.3%	74.9%	72.9%	73.9%
Unknown	58.8%	63.8%	61.4%	69.5%	60.0%	64.8%
**Cigarette smoking**						
Current	60.7%	61.3%	60.9%	67.3%	67.9%	67.6%
Former	67.4%	67.3%	67.4%	76,2%	75.3%	75.8%
Never	69.9%	68.5%	69.2%	76.5%	76.1%	76.3%
Unknown	70.6%	64.1%	67.3%	74.2%	79.9%	77.1%
**Non-study medication**						
ACE-inhibitor or ARB	67.4%	67.6%	67.5%	76.5%	74.8%	75.7%
Aspirin use before screening	70.8%	69.5%	70.1%	79.3%	78.1%	78.7%
Thiazide or related diuretic	68.9%	67.7%	68.3%	76.3%	74.9%	75.7%

Baseline Characteristic	Aspirin Randomization<br>Active (n =7740)	Placebo (n=7740)	Overall (n=15,480)	Omega-3 FAs Randomization<br>Active (n=7740)	Placebo (n=7740)	Overall (n=15,480)
Calcium channel blocker	67.5%	67.1%	67.3%	76.8%	76.5%	76.7%
Statin	69.7%	68.2%	68.9%	76.9%	76.6%	76.7%
**Total cholesterol (mmol/L)**						
<4	71.1%	69.4%	70.3%	78.6%	78.4%	78.5%
≥4 <5	69.2%	68.3%	68.8%	77.1%	76.9%	76.9%
≥5	64.1%	65.2%	64.7%	72.3%	71.1%	72.2%
Not available	65.9%	65.4%	65.7%	73.1%	71.9%	72.5%
**HDL cholesterol (mmol/L)**						
<1	80.7%	80.6%	80.7%	89.5%	87.9%	88.7%
≥1<1.5	68.7%	68.1%	68.4%	78.9%	76.9%	77.9%
≥1.5	69.6%	70.1%	69.8%	77.7%	76.8%	77.2%
Not available	65.9%	65.5%	65.7%	73.1%	72.0%	72.6%
**Non-HDL cholesterol (mmol/L)**						
<2.5	70.8%	70.2%	70.5%	78.9%	78.3%	78.6%
≥2.5 <3.5	70.3%	68.1%	69.2%	77.0%	77.8%	77.4%
≥3.5	64.6%	65.5%	65.1%	74.1%	72.3%	73.2%
Not available	65.9%	65.5%	65.7%	73.1%	72.0%	72.6%
**Glycosylated haemoglobin - HbA1c (IFCC mmol/mol (DCCT %))**						
<48 (6.5)	69.3%	67.4%	68.4%	78.1%	76.1%	77.1%
≥48 (6.5), <64 (8.0)	69.9%	69.7%	69.8%	77.1%	78.3%	77.7%
≥64 (8.0)	67.9%	66.6%	67.3%	75.1%	74.8%	74.9%
Not available	65.9%	65.5%	65.7%	73.1%	71.9%	72.5%
**CKD-EPI estimated GFR (ml/min/1.73m2)** §						
≥90	62.1%	61.5%	61.8%	71.8%	70.7%	71.2%
≥60<90	67.8%	67.4%	67.6%	76.9%	76.2%	76.6%
<60	72.4%	70.9%	71.6%	78.4%	79.0%	78.7%
Not available	65.7%	65.5%	65.7%	73.1%	71.9%	72.5%
**Urinary albumin:creatinine ratio (mg/mmol)**						
<3	69.9%	68.8%	69.4%	77.3%	77.3%	77.3%
≥3	64.6%	65.2%	64.9%	75.6%	75.11%	75.4%
Not available	65.8%	65.4%	65.6%	73.1%	71.7%	72.4%
**Townsend Deprivation Index**						
<-3	68.8%	67.7%	68.2%	77.7%	75.8%	76.7%
≥-3<0	68.9%	68.2%	68.5%	76.1%	75.7%	75.9%
≥0<2	68.6%	68.2%	68.4%	74.5%	75.9%	75.1%
≥2<4	64.3%	63.1%	63.7%	70.5%	72.5%	71.5%
≥4<6	64.3%	63.1%	63.7%	71.7%	70.9%	71.3%
≥6	59.9%	63.5%	61.9%	69.7%	66.9%	68.4%
Unknown	60.5%	82.5%	70.8%	70.9%	72.6%	71.7%
**Ethnic origin**						
White	68.1%	67.4%	67.8%	75.8%	75.2%	75.5%
Indian/Pakistani/Bangladeshi	71.9%	65.6%	68.8%	73.5%	78.3%	75.9%
African/Caribbean	55.0%	57.8%	56.4%	57.9%	68.0%	62.9%
Other/unknown	67.4%	67.6%	67.5%	73.2%	72.9%	73.1%

ACE = angiotensin-converting enzyme; ARB = angiotensin receptor blockers; DCCT = Diabetes Control and Complications Trial; FA = Fatty acids; GFR = Glomerular Filtration Rate; HDL=High-density lipoprotein; IFCC =International Federation of Clinical Chemistry; IQR = Interquartile range; SD = Standard Deviation

*The presence of type 2 diabetes was based on a broad clinical definition involving the participant's age at the diagnosis of diabetes, the use of insulin within one year after diagnosis, and the body-mass index.
† From blood and urine consent forms, generally before randomization.
‡ The body-mass index (the weight in kilograms divided by the square of the height in metres) was based on values for height and weight the participants reported on their randomization questionnaires.
§ Calculated from blood cystatin c concentration using the CKD-EPI formula (Inker LA, Schmid CH, Tighiouart H*, et al.* Estimating Glomerular Filtration Rate from Serum Creatinine and Cystatin C. *New England Journal of Medicine* 2012; 367(1): 20-9)

Criteria used to define definite or probable adherence:

- Definitely adherent = participant reported taking treatment every- or most days during the follow-up period.

- Probably adherent = participant was previously adherent, is still receiving medication and has not reported stopping treatment within the last seven months.

Table S8 Reasons for Stopping Treatment – All Randomized ASCEND Participants

Reasons for Stopping Treatment	Aspirin Randomization						Omega-3 FAs Randomization					
	Active (n=7740)		Placebo (n =7740)		Overall (n=15,480)		Active (n=7740)		Placebo (n=7740)		Overall (n=15,480)	
Participant wishes	1372	(35.8%)	1424	(36.4%)	2796	(36.1%)	1755	(61.0%)	1778	(60.5%)	3533	(60.7%)
Minor bleeding/bruising	115	(3.0%)	72	(1.8%)	187	(2.4%)	8	(0.3%)	4	(0.1%)	12	(0.2%)
Major Bleeding	42	(1.1%)	24	(0.6%)	66	(0.9%)	8	(0.3%)	7	(0.2%)	15	(0.3%)
Aspirin or anti-platelet therapy prescribed by a doctor (clear indication)	630	(16.5%)	748	(19.1%)	1378	(17.8%)	0	(0.0%)	0	(0.0%)	0	(0.0%)
Aspirin or anti-platelet therapy prescribed by a doctor (no clear indication)	257	(6.7%)	241	(6.2%)	498	(6.4%)	0	(0.0%)	0	(0.0%)	0	(0.0%)
Other contraindicated drugs started	249	(6.5%)	267	(6.8%)	516	(6.7%)	15	(0.5%)	12	(0.4%)	27	(0.5%)
Upper GI symptoms/diagnoses/procedures	195	(5.1%)	205	(5.2%)	400	(5.2%)	88	(3.1%)	81	(2.8%)	169	(2.9%)
Lower GI symptoms/diagnoses/procedures	74	(1.9%)	50	(1.3%)	124	(1.6%)	55	(1.9%)	84	(2.9%)	139	(2.4%)
Respiratory or cardiovascular symptoms/diagnoses/procedures	16	(0.4%)	23	(0.6%)	39	(0.5%)	11	(0.4%)	18	(0.6%)	29	(0.5%)
GU symptoms/diagnoses/procedures	13	(0.3%)	13	(0.3%)	26	(0.3%)	11	(0.4%)	14	(0.5%)	25	(0.4%)
Skin symptoms/diagnoses/procedures	17	(0.4%)	14	(0.4%)	31	(0.4%)	14	(0.5%)	9	(0.3%)	23	(0.4%)
Other symptoms/diagnoses/procedures	312	(8.2%)	341	(8.7%)	653	(8.4%)	305	(10.6%)	333	(11.3%)	638	(11.0%)
Administrative reasons (e.g. moved abroad)	237	(6.2%)	257	(6.6%)	494	(6.4%)	279	(9.7%)	266	(9.1%)	545	(9.4%)
Other miscellaneous	32	(0.8%)	22	(0.6%)	54	(0.7%)	34	(1.2%)	33	(1.1%)	67	(1.2%)
Does not have diabetes	23	(0.6%)	29	(0.7%)	52	(0.7%)	23	(0.8%)	21	(0.7%)	44	(0.8%)
Medical advice other	192	(5.0%)	150	(3.8%)	342	(4.4%)	177	(6.1%)	188	(6.4%)	365	(6.3%)
Difficulties swallowing tablets	14	(0.4%)	14	(0.4%)	28	(0.4%)	73	(2.5%)	72	(2.5%)	145	(2.5%)
Difficulties getting tablets out of blister pack	38	(1.0%)	21	(0.5%)	59	(0.8%)	23	(0.8%)	18	(0.6%)	41	(0.7%)
**Total stopped for any reason**	**3828**	**(49.5%)**	**3915**	**(50.6%)**	**7743**	**(50.0%)**	**2879**	**(37.2%)**	**2938**	**(38.0%)**	**5817**	**(37.6%)**

FAs=Fatty Acids; GI=Gastrointestinal; GU=Genitourinary. Reasons were ascertained from free text on questionnaires or following a telephone call to the participant or their managing doctor. The predominant reason was recorded with adverse events taking priority over patient wishes or administrative reasons. Percentages represent the proportion of the total number of participants who stopped treatment for each reason in each column, except for the total row, which represents the proportion of the total population who stopped for any reason. Percentages may not total 100 because of rounding.

Table S9 Heterogeneity of Baseline Characteristics between VFQ Responders and Non-Responders of among those who were Eligible to be sent the VFQ (n=11,301)

Baseline Characteristic	Eligible to be sent the VFQ				P Values†
	Responders (8846)*		Non-responders (n=2455)		
**Age at randomization (years)**					
Mean (SD)	62.5±8.3		62.8±9.0		P=0.11
<60	3339	(37.7%)	901	(36.7%)	
≥60<70	3860	(43.6%)	1024	(41.7%)	
≥70	1647	(18.6%)	530	(21.6%)	P<0.001
**Sex**					
Male	5529	(62.5%)	1550	(63.1%)	
Female	3317	(37.5%)	905	(36.9%)	P = 0.58
**Type of diabetes**					
Type 1	559	(6.3%)	138	(5.6%)	
Type 2	8287	(93.7%)	2317	(94.4%)	P = 0.22
**Duration of diabetes (years)**					
Median (IQR)	7(3-12)		7 (4-13)		P=0.02
≥0<5 years	2918	(33.0%)	751	(30.6%)	
≥5<10 years	2544	(28.8%)	690	(28.1%)	
≥10<20 years	1952	(22.1%)	552	(22.5%)	
≥20 years	1020	(11.5%)	313	(12.7%)	P<0.001
Unknown	412	(4.7%)	149	(6.1%)	
**Diabetes management**					
Diet only	1508	(17.0%)	396	(16.1%)	
Oral hypoglycaemic agent(s) only	5166	(58.4%)	1416	(57.7%)	
Insulin+/- oral hypoglycaemic agents	2172	(24.6%)	643	(26.2%)	P = 0.20
**Participant-reported diabetic retinopathy**					
Yes	1628	(18.4%)	537	(21.9%)	
No	7151	(80.8%)	1895	(77.2%)	P<0.001
Unknown	67	(0.8%)	23	(0.9%)	
**Participant-reported treatment for hypertension**					
Yes	5386	(60.9%)	1534	(62.5%)	
No	3405	(38.5%)	904	(36.8%)	P = 0.14
Unknown	55	(0.6%)	17	(0.7%)	
**Systolic blood pressure (mmHg)**					
Mean (SD)	135.8±14.9		136.3±15.2		P = 0.21
<130	2023	(22.9%)	522	(21.3%)	
≥130<140	1822	(20.6%)	491	(20.0%)	
≥140	2626	(29.7%)	720	(29.3%)	P = 0.82
Unknown	2375	(26.8%)	722	(29.4%)	
**Diastolic blood pressure (mmHg)**					
Mean (SD)	77.4±9.2		77.0±9.6		P=0.11
<75	2371	(26.8%)	650	(26.5%)	
≥75 <85	2788	(31.5%)	753	(30.7%)	
≥85	1311	(14.8%)	326	(13.3%)	P=0.41
Unknown	2376	(26.9%)	726	(29.6%)	
**Body mass index (kg/m^2)**					
Mean (SD)	30.6±6.1		30.9±6.3		P = 0.13
<25	1313	(14.8%)	309	(12.6%)	
≥25, <30	3230	(36.5%)	901	(36.7%)	
≥30<35	2429	(27.5%)	688	(28.0%)	
≥35	1619	(18.3%)	472	(19.2%)	P = 0.05
Unknown	255	(2.9%)	85	(3.5%)	
**Cigarette smoking**					
Current	555	(6.3%)	224	(9.1%)	
Former	3947	(44.6%)	1133	(46.2%)	
Never	4243	(48.0%)	1067	(43.5%)	P<0.001
Unknown	101	(1.1%)	31	(1.3%)	

Baseline Characteristic	Eligible to be sent the VFQ: Responders (8846)*		Eligible to be sent the VFQ: Non-responders (n=2455)		P Values†
**Non-study medication**					
ACE inhibitor or ARB	5115	(57.8%)	1461	(59.5%)	P = 0.05
Aspirin use before screening	3157	(35.7%)	879	(35.8%)	P = 0.93
Thiazide or related diuretic	1639	(18.5%)	465	(18.9%)	P = 0.66
Calcium channel blocker	2088	(23.6%)	605	(24.6%)	P = 0.30
Statin	6769	(76.5%)	1862	(75.8%)	P = 0.50
**Total cholesterol (mmol/L)**					
Mean (SD)	4.1±0.9		4.1±0.9		P = 0.97
<4	2681	(30.3%)	704	(28.7%)	
≥4 <5	2246	(25.4%)	595	(24.2%)	
≥5	817	(9.2%)	226	(9.2%)	P = 0.83
Not available	3102	(35.1%)	930	(37.9%)	
**HDL cholesterol (mmol/L)**					
Mean (SD)	1.3±0.4		1.3±0.4		P=0.02
<1	1222	(13.8%)	343	(14.0%)	
≥1<1.5	3202	(36.2%)	870	(35.4%)	
≥1.5	1310	(14.8%)	307	(12.5%)	P=0.07
Not available	3112	(35.2%)	935	(38.1%)	
**Non-HDL cholesterol (mmol/L)**					
Mean (SD)	2.9±0.8		2.9±0.8		P=0.30
<2.5	2053	(23.2%)	505	(20.6%)	
≥2.5 <3.5	2558	(28.9%)	716	(29.2%)	
≥3.5	1123	(12.7%)	299	(12.2%)	P = 0.14
Not available	3112	(35.2%)	935	(38.1%)	
**Glycosylated haemoglobin - HbA1c**					
IFCC (mmol/mol) mean (SD)	54.0±12.2		55.6±13.5		P<0.001
DCCT (%) mean (SD)	7.1±1.1		7.2±1.2		P<0.001
<48 (6.5)	1957	(22.1%)	496	(20.2%)	
≥48 (6.5), <64 (8.0)	2792	(31.6%)	697	(28.4%)	
≥64 (8.0)	990	(11.2%)	332	(13.5%)	P<0.001
Not available	3107	(35.1%)	930	(37.9%)	
**CKD-EPI estimated GFR (ml/min/1.73m^2)**					
Mean (SD)	87.8±19.8		84.8±20.4		P<0.001
≥90	2902	(32.8%)	671	(27.3%)	
≥60<90	2299	(26.0%)	659	(26.8%)	
<60	540	(6.1%)	194	(7.9%)	P<0.001
Not available	3105	(35.1%)	931	(37.9%)	
**Urinary albumin:creatinine ratio (mg/mmol) ‡**					
Median (IQR)	0.50 (0.00-1.14)		0.59 (0.16-1.40)		P<0.001
<3	5120	(57.9%)	1313	(53.5%)	
≥3	613	(6.9%)	197	(8.0%)	P=0.01
Not available	3113	(35.2%)	945	(38.5%)	
**Townsend Deprivation Index**					
<-3	3101	(35.1%)	764	(31.1%)	
≥-3<0	3588	(40.6%)	930	(37.9%)	
≥0<2	1071	(12.1%)	364	(14.8%)	
≥2<4	625	(7.1%)	244	(9.9%)	
≥4<6	323	(3.7%)	112	(4.6%)	
≥6	118	(1.3%)	37	(1.5%)	P<0.001
Unknown	20	(0.2%)	4	(0.2%)	
**Ethnic origin**					
White	8564	(96.8%)	2346	(95.6%)	
Indian/Pakistani/Bangladeshi	107	(1.2%)	39	(1.6%)	
African/Caribbean	54	(0.6%)	29	(1.2%)	
Other/unknown	121	(1.4%)	41	(1.7%)	P<0.001

DCCT = Diabetes Control and Complications Trial; FAs = Fatty acids; GFR = Glomerular Filtration Rate; HDL=High-density lipoprotein; IFCC =International Federation of Clinical Chemistry; IQR = Interquartile range; SD = Standard Deviation
Figures presented are counts with percentages unless otherwise stated.

*Out of 8846 VFQ respondents, 7 answered a bespoke first page of questions that sought incident eye diagnoses but did not complete the NEI-VFQ-25, and 8839 completed both parts of the form. Separate analyses of the baseline characteristics for the 8839 NEI-VFQ-25 respondents have not been performed because it is unlikely that excluding those 7 participants would make meaningful differences to the average results.
†Formal testing for differences in baseline characteristics between those included in and excluded from the analysis cohort used the Chi-squared test for heterogeneity for categorical variables and grouped continuous variables (including age, duration of diabetes, systolic blood pressure, diastolic blood pressure, body mass index, total cholesterol, HDL-cholesterol, non-HDL cholesterol, HbA1c, eGFR and urinary albumin:creatinine ratio), the Mantel-Haenszel Chi-square test for trend for ordinal variables (i.e. Townsend Deprivation Index), and the t-test or Wilcoxon-Mann-Whitney U test for continuous variables with a normal (summarised by mean±SD) or non-normal (summarised by median and interquartile range) distribution respectively. Categories that were “unknown” or “not available” were excluded.
Percentages may not total 100 because of rounding

Table S10 Duration Follow-up in the Visual Function Questionnaire Respondents

	Aspirin Randomization		Omega-3 FAs Randomization		
	Active (n=4447)	Placebo (n=4399)	Active (n=4417)	Placebo (n=4429)	Overall (n=8846)
Mean(SD) length of follow-up (years)	7.5 (1.4)	7.5 (1.4)	7.5 (1.4)	7.5 (1.4)	7.5 (1.4)
Median (IQR) length of follow-up (years)	7.3 (6.4-8.3)	7.3 (6.4-8.3)	7.3 (6.4-8.7)	7.3 (6.4-8.2)	7.3 (6.4-8.3)
Mean (SD) time from randomization to completing the VFQ (years)	8.6 (1.4)	8.6 (1.4)	8.6 (1.4)	8.6 (1.4)	8.6 (1.4)
Median (IQR) time from randomization to completing the VFQ (years)	8.3 (7.5-9.5)	8.4 (7.5-9.6)	8.4 (7.5-9.6)	8.3 (7.5-9.5)	8.4 (7.5-9.5)

FAs=Fatty Acids; IQR=Interquartile range; SD=Standard deviation; VFQ= Visual Function Questionnaire

Table S11 Reported Definite or Probable Adherence with Study Treatment Stratified by Years Post-Randomization – Visual Function Questionnaire Respondents

	Aspirin Randomization			Omega-3 FAs Randomization		
	Active (%)	Placebo (%)	Overall (%)	Active (%)	Placebo (%)	Overall (%)
**Years post-randomization**						
<3	89.3%	90.3%	89.5%	93.8%	93.2%	93.5%
≥3<5	78.2%	78.3%	78.2%	87.8%	86.9%	87.4%
≥5<7	70.5%	70.6%	70.5%	83.8%	82.6%	83.2%
≥7	62.9%	64.0%	63.5%	79.6%	78.4%	79.0%
**All**	**79.3%**	**79.3%**	**79.3%**	**88.4%**	**87.5%**	**87.9%**

FAs=Fatty Acids.
Criteria used to define definite or probable adherence:

- Definitely adherent = participant reported taking treatment every or most days during the follow-up period.
- Probably adherent = participant was previously adherent, is still receiving medication and has not reported stopping treatment within the last seven months.

Table S12 Use of Anti-platelet or Anti-coagulant Therapy in the Aspirin Arm during Follow-Up – Visual Function Questionnaire Respondents

	Aspirin Randomization				
	Active (n=4447)			Placebo (n=4399)	
	Study aspirin	Study aspirin or non-study anti-platelet	Study aspirin or non-study anti-platelet agent or anti-coagulant	Non-study anti-platelet	Non-study anti-platelet agent or anti-coagulant
**Years post-randomization**					
<3	89.5%	92.1%	92.5%	3.3%	3.8%
≥3<5	78.2%	84.6%	86.2%	7.3%	8.9%
≥5<7	70.5%	79.4%	81.9%	9.8%	12.9%
≥7	63.5%	75.9%	78.7%	12.6%	16.4%
**All**	**79.3%**	**85.5%**	**86.9%**	**6.9%**	**8.6%**

Table S13 Study Average Reported Definite or Probable Adherence in each Treatment Arm, Overall and by Baseline Characteristics – Visual Function Questionnaire Respondents

	**Aspirin Randomization**			**Omega-3 FAs Randomization**		
**Baseline Characteristic**	**Active (n =4447)**	**Placebo (n=4399)**	**Overall (n=8846)**	**Active (n=4417)**	**Placebo (n=4429)**	**Overall (n=8846)**
**Age at randomization (years)**						
<60	82.7%	82.3%	82.5%	89.1%	88.8%	88.9%
≥60<70	77.9%	79.3%	78.6%	88.2%	87.4%	87.8%
≥70	74.9%	73.6%	74.3%	87.1%	84.9%	86.0%
**Sex**						
Male	80.3%	80.8%	80.5%	89.8%	89.4%	89.6%
Female	77.6%	77.2%	77.4%	86.1%	84.3%	85.2%
**Type of diabetes***						
Type 1	82.4%	81.3%	81.9%	89.7%	88.3%	89.1%
Type 2	79.0%	79.3%	79.2%	88.3%	87.4%	87.9%
**Duration of diabetes (years)**						
≥0<5 years	80.9%	80.5%	80.7%	89.4%	88.4%	88.9%
≥5<10 years	77.6%	78.8%	78.2%	87.7%	85.7%	86.7%
≥10<20 years	79.5%	79.4%	79.4%	88.6%	88.8%	88.7%
≥20 years	79.7%	79.2%	79.5%	88.3%	87.8%	88.0%
Unknown	76.3%	76.3%	76.3%	85.2%	85.5%	85.3%
**Diabetes management**						
Diet only	76.6%	78.3%	77.5%	88.8%	84.4%	86.5%
Oral hypoglycaemic agent(s) only	79.9%	79.3%	79.6%	88.2%	88.3%	88.3%
Insulin +/- oral hypoglycaemic agent(s)	79.4%	80.6%	79.9%	88.5%	87.8%	88.2%
**Participant-reported diabetic retinopathy**						
Yes	77.5%	79.1%	78.3%	88.5%	87.9%	88.2%
No	79.6%	79.6%	79.6%	88.4%	87.4%	87.9%
**Participant-reported treatment for hypertension**						
Yes	78.2%	79.1%	78.6%	88.3%	87.3%	87.8%
No	80.8%	79.9%	80.3%	88.5%	87.8%	88.2%
**Systolic blood pressure (mmHg) †**						
<130	79.4%	80.8%	80.1%	88.5%	88.0%	88.2%
≥130<140	81.9%	79.0%	80.4%	89.7%	88.4%	89.1%
≥140	78.0%	79.2%	78.6%	88.5%	88.2%	88.3%
Unknown	78.6%	78.9%	78.8%	87.2%	85.6%	86.4%
**Diastolic blood pressure (mmHg) †**						
<75	78.2%	78.1%	78.1%	87.5%	87.1%	87.3%
≥75 <85	80.8%	80.4%	80.6%	89.7%	88.5%	89.1%
≥85	79.4%	80.7%	80.1%	89.3%	89.7%	89.5%
Unknown	78.6%	78.9%	78.7%	87.3%	85.6%	86.4%
**Body mass index (kg/m^2) ‡**						
<25	80.5%	81.5%	80.9%	89.6%	88.0%	88.8%
≥25, <30	78.4%	79.9%	79.1%	88.3%	87.7%	88.0%
≥30<35	79.9%	79.3%	79.6%	88.1%	88.4%	88.2%
≥35	79.1%	77.4%	78.3%	88.2%	85.9%	87.1%
Unknown	76.5%	80.4%	78.6%	86.1%	73.6%	80.5%
**Cigarette smoking**						
Current	77.4%	78.2%	77.8%	85.3%	86.7%	86.1%
Former	78.5%	79.5%	78.9%	88.4%	88.2%	88.3%
Never	80.1%	79.8%	79.9%	88.8%	86.9%	87.9%
Unknown	83.4%	70.9%	76.9%	89.6%	87.9%	88.8%

Baseline Characteristic	Aspirin Randomization Active (n =4447)	Placebo (n=4399)	Overall (n=8846)	Omega-3 FAs Randomization Active (n=4417)	Placebo (n=4429)	Overall (n=8846)
**Non-study medication**						
ACE-inhibitor or ARB	78.3%	79.7%	79.0%	88.7%	87.7%	88.2%
Aspirin use before screening	80.0%	80.2%	80.1%	90.1%	88.9%	89.6%
Thiazide or related diuretic	78.6%	79.3%	78.9%	88.2%	86.9%	87.5%
Calcium channel blocker	77.7%	77.7%	77.7%	89.2%	87.9%	88.5%
Statin	80.3%	79.9%	80.1%	88.8%	88.2%	88.5%
**Total cholesterol (mmol/L)**						
<4	80.5%	78.9%	79.7%	88.6%	89.3%	88.9%
≥4 <5	79.6%	79.9%	79.8%	89.9%	87.5%	88.7%
≥5	77.0%	76.7%	76.9%	86.9%	85.3%	86.1%
Not available	78.5%	80.3%	79.4%	87.5%	86.5%	87.0%
**HDL cholesterol (mmol/L)**						
<1	80.8%	78.7%	79.8%	90.5%	89.8%	90.2%
≥1<1.5	78.8%	78.6%	78.8%	88.7%	87.4%	88.0%
≥1.5	80.5%	80.2%	80.4%	87.9%	87.9%	87.9%
Not available	78.5%	80.3%	79.4%	87.4%	86.6%	86.9%
**Non-HDL cholesterol (mmol/L)**						
<2.5	80.5%	80.2%	80.3%	89.3%	88.8%	89.1%
≥2.5 <3.5	80.2%	78.7%	79.5%	88.9%	88.4%	88.6%
≥3.5	77.1%	77.6%	77.3%	88.2%	85.6%	86.9%
Not available	78.5%	80.3%	79.4%	87.4%	86.6%	86.9%
**Glycosylated haemoglobin - HbA1c (IFCC mmol/mol (DCCT %))**						
<48 (6.5)	78.5%	78.5%	78.5%	89.5%	87.2%	88.3%
≥48 (6.5), <64 (8.0)	79.9%	79.4%	79.6%	88.4%	88.7%	88.5%
≥64 (8.0)	81.6%	79.1%	80.3%	88.9%	87.9%	88.5%
Not available	78.5%	80.2%	79.3%	87.5%	86.5%	87.0%
**CKD-EPI estimated GFR (ml/min/1.73m2)** §						
≥90	73.1%	71.8%	72.5%	84.3%	83.6%	83.9%
≥60<90	77.4%	77.8%	77.6%	88.4%	87.1%	87.8%
<60	82.5%	81.2%	81.9%	90.0%	89.5%	89.7%
Not available	78.5%	80.3%	79.4%	87.5%	86.5%	87.0%
**Urinary albumin:creatinine ratio (mg/mmol)**						
<3	80.1%	79.6%	79.8%	88.7%	88.3%	88.5%
≥3	76.7%	74.2%	75.4%	89.7%	85.9%	87.9%
Not available	78.4%	80.3%	79.4%	87.5%	86.5%	87.0%
**Townsend Deprivation Index**						
<-3	78.8%	78.8%	78.8%	88.8%	87.7%	88.2%
≥-3<0	78.4%	79.4%	78.9%	87.8%	86.8%	87.3%
≥0<2	81.4%	80.8%	81.1%	88.7%	88.5%	88.6%
≥2<4	81.3%	79.3%	80.3%	87.1%	88.0%	87.6%
≥4<6	82.9%	80.09%	81.9%	92.1%	89.7%	90.9%
≥6	80.3%	78.8%	79.5%	87.6%	86.7%	87.2%
Unknown	74.2%	94.2%	86.1%	99.2%	86.9%	91.9%
**Ethnic origin**						
White	79.2%	79.5%	79.3%	88.5%	87.4%	87.9%
Indian/Pakistani/Bangladeshi	81.2%	78.6%	79.9%	83.2%	88.8%	86.0%
African/Caribbean	70.2%	80.1%	75.2%	88.3%	91.7%	90.3%
Other/unknown	88.5%	77.0%	82.6%	86.2%	91.4%	88.2%

ACE = angiotensin-converting enzyme; ARB = angiotensin receptor blockers; DCCT = Diabetes Control and Complications Trial; FA = Fatty acids; GFR = Glomerular Filtration Rate; HDL=High-density lipoprotein; IFCC =International Federation of Clinical Chemistry; IQR = Interquartile range; SD = Standard Deviation

*The presence of type 2 diabetes was based on a broad clinical definition involving the participant's age at the diagnosis of diabetes, the use of insulin within one year after diagnosis, and the body-mass index.
† From blood and urine consent forms, generally before randomization.
‡ The body-mass index (the weight in kilograms divided by the square of the height in metres) was based on values for height and weight the participants reported on their randomization questionnaires.

§ Calculated from blood cystatin c concentration using the CKD-EPI formula (Inker LA, Schmid CH, Tighiouart H*, et al.* Estimating Glomerular Filtration Rate from Serum Creatinine and Cystatin C. *New England Journal of Medicine* 2012; 367(1): 20-9)

Criteria used to define definite or probable adherence:

- Definitely adherent = participant reported taking treatment every or most days during the follow-up period.
- Probably adherent = participant was previously adherent, is still receiving medication and has not reported stopping treatment within the last seven months.

Table S14 Reasons for Stopping Treatment – Visual Function Questionnaire Respondents

Reasons for stopping treatment	Aspirin Randomization Active (n=4447)		Aspirin Randomization Placebo (n =4399)		Aspirin Randomization Overall (n=8846)		Omega-3 FAs Randomization Active (n =4417)		Omega-3 FAs Randomization Placebo (n =4429)		Omega-3 FAs Randomization Overall (n=8846)	
Participant wishes	406	(27.6%)	429	(29.6%)	835	(28.6%)	486	(65.4%)	550	(67.0%)	1036	(66.2%)
Minor bleeding/bruising	59	(4.0%)	46	(3.2%)	105	(3.6%)	4	(0.5%)	3	(0.4%)	7	(0.4%)
Major Bleeding	22	(1.5%)	7	(0.5%)	29	(1.0%)	3	(0.4%)	2	(0.2%)	5	(0.3%)
Aspirin or anti-platelet therapy prescribed by a doctor (clear indication)	347	(23.6%)	381	(26.3%)	728	(24.9%)	0	(0.0%)	0	(0.0%)	0	(0.0%)
Aspirin or anti-platelet therapy prescribed by a doctor (no clear indication	138	(9.4%)	126	(8.7%)	264	(9.0%)	0	(0.0%)	0	(0.0%)	0	(0.0%)
Other contraindicated drugs started	140	(9.5%)	152	(10.5%)	292	(10.0%)	7	(0.9%)	10	(1.2%)	17	(1.1%)
Upper GI symptoms/diagnoses/procedures	107	(7.3%)	101	(7.0%)	208	(7.1%)	42	(5.7%)	23	(2.8%)	65	(4.2%)
Lower GI symptoms/diagnoses/procedures	37	(2.5%)	26	(1.8%)	63	(2.2%)	27	(3.6%)	40	(4.9%)	67	(4.3%)
Respiratory or cardiovascular symptoms/diagnoses/procedures	2	(0.1%)	9	(0.6%)	11	(0.4%)	2	(0.3%)	1	(0.1%)	3	(0.2%)
GU symptoms/diagnoses/procedures	5	(0.3%)	6	(0.4%)	11	(0.4%)	4	(0.5%)	6	(0.7%)	10	(0.6%)
Skin symptoms/diagnoses/procedures	8	(0.5%)	6	(0.4%)	14	(0.5%)	4	(0.5%)	3	(0.4%)	7	(0.4%)
Other symptoms/diagnoses/procedures	83	(5.6%)	73	(5.0%)	156	(5.3%)	49	(6.6%)	61	(7.4%)	110	(7.0%)
Administrative reasons (e.g. moved abroad)	3	(0.2%)	4	(0.3%)	7	(0.2%)	2	(0.3%)	5	(0.6%)	7	(0.4%)
Other miscellaneous	4	(0.3%)	7	(0.5%)	11	(0.4%)	6	(0.8%)	6	(0.7%)	12	(0.8%)
Does not have diabetes	13	(0.9%)	17	(1.2%)	30	(1.0%)	8	(1.1%)	14	(1.7%)	22	(1.4%)
Medical advice other	75	(5.1%)	42	(2.9%)	117	(4.0%)	58	(7.8%)	56	(6.8%)	114	(7.3%)
Difficulties swallowing tablets	6	(0.4%)	6	(0.4%)	12	(0.4%)	30	(4.0%)	35	(4.3%)	65	(4.2%)
Difficulties getting tablets out of blister pack	17	(1.2%)	9	(0.6%)	26	(0.9%)	11	(1.5%)	6	(0.7%)	17	(1.1%)
**Total stopped for any reason**	**1472**	**(33.1%)**	**1447**	**(32.9%)**	**2919**	**(33.0%)**	**743**	**(16.8%)**	**821**	**(18.5%)**	**1564**	**(17.7%)**

FAs=Fatty Acids; GI=Gastrointestinal; GU=Genitourinary; Reasons were ascertained from free text on questionnaires or following a telephone call to the participant or their managing doctor. The predominant reason was recorded with adverse events taking priority over patient wishes or administrative reasons. Percentages represent the proportion of the total number of participants who stopped treatment for each reason in each column, except for the total row, which represents the proportion of the total population who stopped for any reason. Percentages may not total 100 because of rounding.

Table S15 Source of Age-Related Macular Degeneration Events

Confirmed or unrefuted AMD events reported on ASCEND follow-up questionnaires	VFQ form returned			No VFQ	Total
	AMD reported on VFQ	AMD not reported on VFQ	Total		
Yes	33	13	46	34	80
No	180	8620	8800	6600	15400
**Total**	213	13	8846	6634	15480

AMD= Age-Related Macular Degeneration; VFQ=Visual Function Questionnaire

Table S16 Effects of Aspirin and Omega-3 Fatty Acids on Age-Related Macular Degeneration Events Stratified by the Other Treatment in the Factorial Design

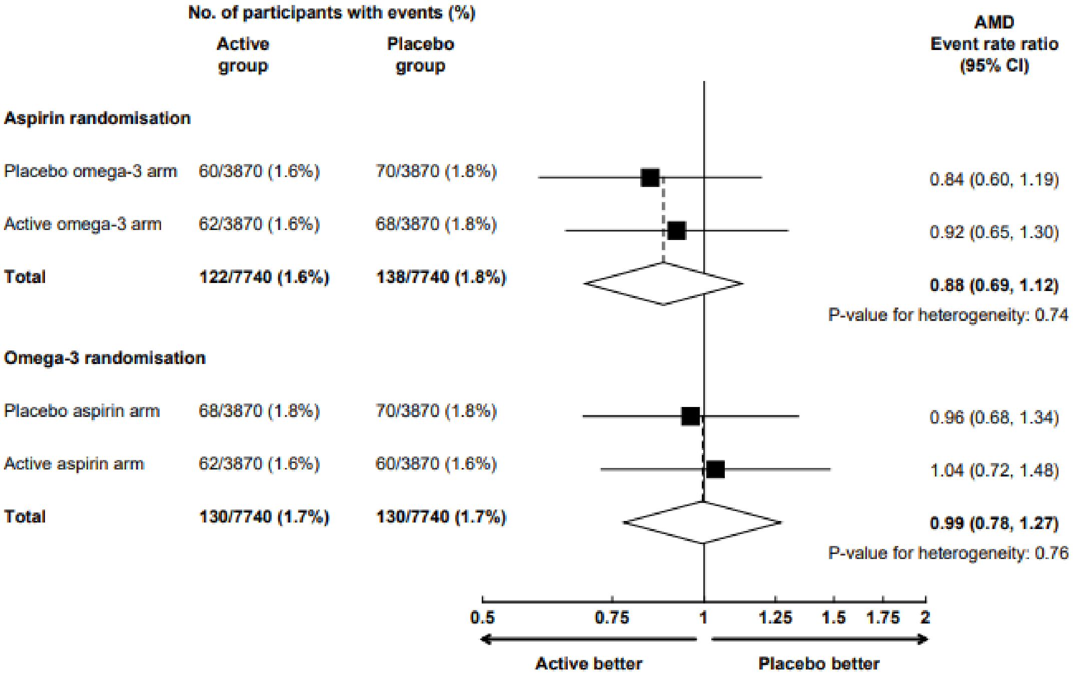

ASCEND Follow-Up Questionnaire

ASCEND: Follow-up Questionnaire

INSTRUCTIONS FOR COMPLETION:
Please complete the questionnaire using blue or black ink.
Please place a cross in the appropriate box, e.g. Yes [X] No [ ]
(If you make a mistake, fill the entire box and mark the correct box, e.g. Yes ■ No [X])
OR write clearly in the appropriate boxes, e.g. 20 / 04 / 2004 (Day / Month / Year)

1. Contact and GP Details

Please check that these contact details are still correct. If not, then please telephone 0800 585323 and provide the correct information. Please quote the reference number from the covering letter on the front of this questionnaire.

Your details:	***Alternative contact:***	***GP details:***
Rev Frederick Jones<br>99 The Street<br>Littletown<br>Toyshire<br>WW1 1PY	Mrs Jane Jones<br>The House<br>Bigtown<br>Toyshire<br>WW3 2MM	Dr Roger Smith<br>The Surgery<br>Toytown<br>Toyshire<br>WW2 8XX
Home Tel.: 01456 789 101<br>Daytime Tel.: 01456 987654	Tel.: 01234 765432	

2. ASCEND Medication

2.1. Please indicate how regularly you have taken your ASCEND medication during the last 6 months:

F-190504

	White Tablets (aspirin/placebo)	Red Capsules (one or other natural oil)
Every day	[ ]	[ ]
Most days	[ ]	[ ]
Only occasionally	[ ]	[ ]
Never	[ ]	[ ]

*Please cross **ONE** box only in each column*

2.2 Are you willing to continue taking the **white** (aspirin/placebo) ASCEND tablets?
Yes [ ] No [ ]
If **No**, please tell us why:

2.3 Are you willing to continue taking the **red** (one or other natural oil) ASCEND capsules?
Yes [ ] No [ ]
If **No**, please tell us why:

+

3. Other Current Medication

3.1 Do you currently take any of the following **regularly** (i.e. more than one day per week)?

		Yes	No
a)	Warfarin (Marevan), Acenocoumarol (Nicoumalone, Sinthrome) or Phenindione	[ ]	[ ]
b)	Aspirin, prescribed or over-the-counter (e.g. Anadin, Caprin, Disprin, Imazin, PostMI). *Do not include your ASCEND study tablets.*	[ ]	[ ]
c)	Clopidogrel (Plavix)	[ ]	[ ]
d)	Dipyridamole (Persantin, Persantin Retard or Asasantin Retard)	[ ]	[ ]

*Please cross **ONE** box only for each question*

Follow-up Quest [V2_190504] 2 Reference 123-4567

Need help completing this form? Please call Freefone 0800 585323 +

4. Medical Events

4.1 Since completing your last questionnaire on **1 February 2003** have you had ANY of the following?
(If **Yes**, please give the date and the name and town of the hospital you attended)

a) **Heart attack** Yes [ ] No [ ] Day / Month / Year
Name and town of hospital attended:

b) **Admission to hospital with angina or any chest pains** Yes [ ] No [ ] Day / Month / Year
Name and town of hospital attended:

c) **Stroke** Yes [ ] No [ ] Day / Month / Year
Name and town of hospital attended:

d) **Ministroke (sometimes called TIA)** Yes [ ] No [ ] Day / Month / Year
Name and town of hospital attended:

e) **Coronary artery bypass operation (CABG or "cabbage")** Yes [ ] No [ ] Day / Month / Year
Name and town of hospital attended:

f) **Coronary angioplasty ("balloon", "stent" insertion or PTCA)** Yes [ ] No [ ] Day / Month / Year
Name and town of hospital attended:

g) **Other arterial surgery or angioplasty (e.g. leg bypass)** Yes [ ] No [ ] Day / Month / Year
Name and town of hospital attended:

h) **Cancer (e.g. skin, breast, lung, bowel etc)** Yes [ ] No [ ] Day / Month / Year
Type of cancer:
Name and town of hospital attended:

i) **Bleeding for which you saw a doctor (e.g. serious nose bleed, bleeding in the eye)** ***Do not include bleeding as a result of an accident.*** Yes [ ] No [ ] Day / Month / Year
Site in body of bleeding:
Were you admitted to hospital? Yes [ ] No [ ]
Name and town of hospital attended:

Follow-up Quest [V2_190504] 3 Reference 123-4567 +

Need help completing this form? Please call Freefone 0800 585323

5. Other Serious Illnesses or Hospital Admissions

5.1 If since completing your last questionnaire on **1 February 2003** you have had any other serious illness or admission to hospital (e.g. pneumonia, day surgery, laser treatment to the eye) please give details of the illness or surgery, the date, and the name and town of the hospital you attended.

Details of illness or admission:
Name and town of hospital attended:
Date: Day / Month / Year

Details of illness or admission:
Name and town of hospital attended:
Date: Day / Month / Year

Details of illness or admission:
Name and town of hospital attended:
Date: Day / Month / Year

Details of illness or admission:
Name and town of hospital attended:
Date: Day / Month / Year

6. Personal Details

6.1 Please give your date of birth: Day / Month / Year

Thank you for completing the questionnaire.
Please SIGN and DATE the form below using blue or black ink.

Signature:
& **PRINTED** name: Today's date: Day / Month / Year

Please check that you have answered every question, and signed and dated the form. Return the completed form in the Freepost envelope provided (no stamps needed) to: +

ASCEND, FREEPOST NAT13900, Harkness Building, Radcliffe Infirmary, OXFORD, OX2 6BR

If you have any questions about the study, please contact the coordinating centre in Oxford on FREEFONE: 0800 585323 (preferably during office hours 9 am – 5 pm, Monday to Friday)

Thank you for your continued participation in ASCEND

Follow-up Quest [V2_190504] 4 Reference 123-4567

British Heart Foundation

ASCEND

A Study of Cardiovascular Events iN Diabetes

ASCEND
Clinical Trial Service Unit
Harkness Building
Radcliffe Infirmary
Oxford
OX2 6HE

Office telephone: 01865 404888
Office fax: 01865 404871
Freefone: 0800 585323
e-mail: ascend@ctsu.ox.ac.uk
Website: www.ctsu.ox.ac.uk/ascend

Attachment 7: Amendments to B22-2
Participant Follow-up letter and Questionnaire

7 August 2003

Rev Frederick Jones
99 The Street
Littletown
Toyshire
WW1 PY1

+

Ref: 123-4567

Dear Rev Jones

ASCEND: A Study of Cardiovascular Events iN Diabetes

Thank you for your continued participation in ASCEND and your commitment to research into diabetes. On the back of this letter is your regular questionnaire. We would be grateful if you would complete it and return it promptly in the Freepost envelope enclosed. We cannot emphasise enough how important it is that we receive this information regularly from each of the 10,000 participants in ASCEND. We shall be sending you a new supply of study treatment automatically when it is required.

Please let us know if you have any questions or need help completing the questionnaire, by telephoning Freefone: 0800 585323.

Thank you for your continued support of ASCEND and for completing the regular questionnaires. We are grateful for your help with finding ways of preventing the complications of diabetes.

Yours sincerely

Dr Jane Armitage Dr Louise Bowman
Study Coordinators

Enc: Freepost envelope

+

Follow-up Quest [V2_190504] 1 Reference 123-4567 MRC Medical Research Council

ASCEND-Eye Visual Function Questionnaire

PART 3 – RESPONSES TO VISION PROBLEMS   Pt ID: A123-4567   K2/780/A425-9454/G

The next questions are about how things you do may be affected by your vision. For each one, please cross the box to indicate whether for you the statement is true for you all, most, some, a little, or none of the time.

(please cross **ONE** box only for **EACH** question)	All of the time	Most of the time	Some of the time	A little of the time	None of the time
3.17 Do you accomplish less than you would like because of your vision?	☐	☐	☐	☐	☐
3.18 Are you limited in how long you can work or do other activities because of your vision?	☐	☐	☐	☐	☐
3.19 How much does pain or discomfort in or around your eyes, for example, burning, itching or aching, keep you from doing what you'd like to be doing?	☐	☐	☐	☐	☐

For each of the following statements, please cross the box to indicate whether for you the statement is definitely true, mostly true, mostly false or definitely false or you are not sure.

(please cross **ONE** box only for **EACH** question)	Definitely true	Mostly true	Not sure	Mostly false	Definitely false
3.20 I stay home most of the time because of my eyesight.	☐	☐	☐	☐	☐
3.21 I feel frustrated a lot of the time because of my eyesight.	☐	☐	☐	☐	☐
3.22 I have much less control over what I do, because of my eyesight.	☐	☐	☐	☐	☐
3.23 Because of my eyesight, I have to rely too much on what other people tell me.	☐	☐	☐	☐	☐
3.24 I need a lot of help from others because of my eyesight.	☐	☐	☐	☐	☐
3.25 I worry about doing things that will embarrass myself or others, because of my eyesight.	☐	☐	☐	☐	☐

4. Personal Details

4.1 Please reconfirm your date of birth: __ __ / __ __ / 1 9 __ __ (Day / Month / Year)

Thank you for completing the questionnaire.
Please SIGN and DATE the form below using blue or black ink.

Signature:

& PRINTED name: Today's date: __ __ / __ __ / 2 0 __ __ (Day / Month / Year)

Please check that you have answered **every** question, and **signed and dated** the form.
Return the completed questionnaire in the **Freepost** envelope provided **(no stamps needed)** to:

Freepost RLUJ-TKES-SURB, ASCEND, Richard Doll Building, University of Oxford, Old Road Campus, Headington, Oxford, OX3 7LF

If you have any questions about the study, please contact the coordinating centre in Oxford on **FREEFONE: 0800 585323** (preferably during office hours 9 am - 5 pm, Monday to Friday)

Thank you for your participation in ASCEND

ASCEND-EYE Visual Function Questionnaire [V1.1_08-08-2017]   4

ASCEND-EYE: Visual Function Questionnaire

INSTRUCTIONS FOR COMPLETION:

Please complete the questionnaire in BLOCK CAPITALS using blue or black ink.
Please place a cross in the appropriate box, e.g. Yes ☒ No ☐
(If you make a mistake, fill the entire box and mark the correct box, e.g. Yes ■ No ☒)
OR write clearly in the appropriate boxes, e.g. 2 6 / 0 1 / 2 0 1 7 (Day / Month / Year)

Please complete all the questions **as if you were wearing your glasses or contact lenses** (if any).
Please answer **every** question (unless you are asked to skip questions because they don't apply to you).

1. Participant Name and Address

Mr Thomas WHITE
24 Raspberry Road, Gardentown
Gardenshire, GA3 5TR

Form ID: A425-9454
Participant Study Ref: A123-4567

2. Eye Events

2.1 Have you had ANY of the following?
(If **Yes**, please give the date you were first diagnosed and the name and town of the hospital you first attended).

a) **Cataract**   Yes ☐ No ☐   __ __ / __ __ / __ __ __ __ (Day / Month / Year)
Name and town of hospital attended:

b) **Age-related macular degeneration**   Yes ☐ No ☐   __ __ / __ __ / __ __ __ __ (Day / Month / Year)
Name and town of hospital attended:

c) **Glaucoma**   Yes ☐ No ☐   __ __ / __ __ / __ __ __ __ (Day / Month / Year)
Name and town of hospital attended:

d) **Retinal vein thrombosis**   Yes ☐ No ☐   __ __ / __ __ / __ __ __ __ (Day / Month / Year)
Name and town of hospital attended:

e) **Other eye problems**

Name and town of hospital attended:

Date: __ __ / __ __ / __ __ __ __ (Day / Month / Year)

Pt ID: A123-4567   K2/780/A425-9454G   8th September 2017

ASCEND-EYE Visual Function Questionnaire [V1.1_08-08-2017]   1

Need help completing this form? Please call Freefone 0800 585323

3. National Eye-Institute Visual Functioning Questionnaire - 25

PART 1 – GENERAL HEALTH AND VISION (please cross **ONE** box only for **EACH** question)

3.1 In general, would you say your overall health is:
Excellent: ☐ Very good: ☐ Good: ☐ Fair: ☐ Poor: ☐

3.2 At the present time, would you say your eyesight using both eyes (with glasses or contact lenses, if you wear them) is excellent, good, fair, poor, or very poor or are you completely blind?
Excellent: ☐ Good: ☐ Fair: ☐ Poor: ☐ Very poor: ☐ Completely blind: ☐

3.3 How much of the time do you worry about your eyesight?
None of the time: ☐ A little of the time: ☐ Some of the time: ☐ Most of the time: ☐ All of the time: ☐

3.4 How much pain or discomfort have you had in and around your eyes (for example, burning, itching, or aching)? Would you say it is:
None: ☐ Mild: ☐ Moderate: ☐ Severe: ☐ Very severe: ☐

PART 2 – DIFFICULTY WITH ACTIVITIES (please cross **ONE** box only for **EACH** question)

The next questions are about how much difficulty, if any, you have doing certain activities, wearing your glasses or contact lenses if you use them for that activity.

3.5 How much difficulty do you have reading ordinary print in newspapers? Would you say you have:
No difficulty at all: ☐ A little difficulty: ☐ Moderate difficulty: ☐ Extreme difficulty: ☐ Stopped doing this because of your eyesight: ☐ Stopped doing this for other reasons or not interested in doing this: ☐

3.6 How much difficulty do you have doing work or hobbies that require you to see well up close, such as cooking, sewing, fixing things around the house or using hand tools? Would you say:
No difficulty at all: ☐ A little difficulty: ☐ Moderate difficulty: ☐ Extreme difficulty: ☐ Stopped doing this because of your eyesight: ☐ Stopped doing this for other reasons or not interested in doing this: ☐

3.7 Because of your eyesight, how much difficulty do you have finding something on a crowded shelf?
No difficulty at all: ☐ A little difficulty: ☐ Moderate difficulty: ☐ Extreme difficulty: ☐ Stopped doing this because of your eyesight: ☐ Stopped doing this for other reasons or not interested in doing this: ☐

3.8 How much difficulty do you have reading street signs or the names of shops?
No difficulty at all: ☐ A little difficulty: ☐ Moderate difficulty: ☐ Extreme difficulty: ☐ Stopped doing this because of your eyesight: ☐ Stopped doing this for other reasons or not interested in doing this: ☐

3.9 Because of your eyesight, how much difficulty do you have going down steps, stairs, or kerbs in dim light or at night?
No difficulty at all: ☐ A little difficulty: ☐ Moderate difficulty: ☐ Extreme difficulty: ☐ Stopped doing this because of your eyesight: ☐ Stopped doing this for other reasons or not interested in doing this: ☐

V-080817

ASCEND-EYE Visual Function Questionnaire [V1.1_08-08-2017]   2

Pt ID: A123-4567   K2/780/A425-9454G   8th September 2017

3.10 Because of your eyesight, how much difficulty do you have noticing objects off to the side while you are walking along?
No difficulty at all: ☐ A little difficulty: ☐ Moderate difficulty: ☐ Extreme difficulty: ☐ Stopped doing this because of your eyesight: ☐ Stopped doing this for other reasons or not interested in doing this: ☐

3.11 Because of your eyesight, how much difficulty do you have seeing how people react to things you say?
No difficulty at all: ☐ A little difficulty: ☐ Moderate difficulty: ☐ Extreme difficulty: ☐ Stopped doing this because of your eyesight: ☐ Stopped doing this for other reasons or not interested in doing this: ☐

3.12 Because of your eyesight, how much difficulty do you have picking out and matching your own clothes?
No difficulty at all: ☐ A little difficulty: ☐ Moderate difficulty: ☐ Extreme difficulty: ☐ Stopped doing this because of your eyesight: ☐ Stopped doing this for other reasons or not interested in doing this: ☐

3.13 Because of your eyesight, how much difficulty do you have visiting with people in their homes, at parties, or in restaurants?
No difficulty at all: ☐ A little difficulty: ☐ Moderate difficulty: ☐ Extreme difficulty: ☐ Stopped doing this because of your eyesight: ☐ Stopped doing this for other reasons or not interested in doing this: ☐

3.14 Because of your eyesight, how much difficulty do you have going out to see films, plays, or sports events?
No difficulty at all: ☐ A little difficulty: ☐ Moderate difficulty: ☐ Extreme difficulty: ☐ Stopped doing this because of your eyesight: ☐ Stopped doing this for other reasons or not interested in doing this: ☐

3.15 Are you currently driving, at least once in a while? Yes ☐ No ☐

3.15a IF NO: have you never driven a car or have you given up driving?
Never drove: ☐ → Skip to Part 3, Q 3.17 on page 4
Gave up: ☐

3.15b IF YOU GAVE UP DRIVING: Was that mainly because of your eyesight, mainly for some other reason, or because of both your eyesight and other reasons?
Mainly eyesight: ☐ Mainly other reason: ☐ Both eyesight and other reasons: ☐ → Skip to Part 3, Q 3.17 on page 4

3.15c IF CURRENTLY DRIVING: How much difficulty do you have driving during the daytime in familiar places? Would you say you have:
No difficulty at all: ☐ A little difficulty: ☐ Moderate difficulty: ☐ Extreme difficulty: ☐

3.16 How much difficulty do you have driving at night? Would you say you have:
No difficulty at all: ☐ A little difficulty: ☐ Moderate difficulty: ☐ Extreme difficulty: ☐ Stopped doing this because of your eyesight: ☐ Stopped doing this for other reasons or not interested in doing this: ☐

3.16A How much difficulty do you have driving in difficult conditions, such as in bad weather, during rush hour, on the motorway, or in city traffic? Would you say you have:
No difficulty at all: ☐ A little difficulty: ☐ Moderate difficulty: ☐ Extreme difficulty: ☐ Stopped doing this because of your eyesight: ☐ Stopped doing this for other reasons or not interested in doing this: ☐

3
