# Supplementary material for: Effects of aspirin and omega-3 fatty acids on age-related macular degeneration in ASCEND-Eye: a randomised placebo-controlled trial in a population with diabetes
Source: BMJ Open. 2025 Feb 26;15(2):e090605. doi: 10.1136/bmjopen-2024-090605 (PMC11865773; doi:10.1136/bmjopen-2024-090605)
Supplement: Supplementary file 2 [file bmjopen-2024-090605.reviewer_comments.pdf]

PEER REVIEW HISTORY

BMJ Open publishes all reviews undertaken for accepted manuscripts. Reviewers are asked to complete a checklist review form and are provided with free text boxes to elaborate on their assessment. These free text comments are reproduced below.

ARTICLE DETAILS

Title (Provisional)

The Effects of Aspirin and Omega-3 Fatty Acids on Age-Related Macular Degeneration in ASCEND-Eye, A Randomised Placebo Controlled Trial in a Population with Diabetes

Authors

Sammons, Emily; Bowman, Louise; Stevens, William; Buck, Georgina; Hammami, Imen; Parish, Sarah; Armitage, Jane; ASCEND Study Collaborative Group, ASCEND Study Collaborative Group

---

VERSION 1 - REVIEW

**Reviewer**	**1**
**Name**	**Moinuddin, Mohammed**
**Affiliation**	**University of Central Lancashire, School of Medicine and Dentistry**
**Date**	**16-Oct-2024**
**COI**	**None**

Statistical reviewer's comment

The authors presented results from a randomised control trial to assess the effect of Aspirin and Omega-3 fatty acids on age-related macular degeneration. A well-designed RCT with minimised randomisation and masked however the results are negative. It was not an interesting reading due to excessive reference to their other works from the same trial. To me, this piece does not add much value to the science literature as most of the aspects of the study are already published elsewhere. The only addition is that aspirin and omega-3 fatty acids did not help the patients under study. Moreover, the effect measure and statistical analysis were not up to the mark. My specific observations are below.

Comment 1 – I wonder if the outcome AMD was measured from a single eye or both or any? If both eyes were measured there is a possibility of correlated measures. What did the author think about this? Needs clarification.

Comment 2 – I wonder why the statistical analysis section did not discuss what effect sizes were and how they were measured. Of course, while I read the results it is visible that the rate ratio was measured. I would suggest describing this in the statistical analysis section. Also, I was wondering why the interaction effect of the two interventions was not measured. It is not even discussed. To me, this analysis of just reporting a rate ratio with a 95% confidence interval is not complete unless a proper modelling exercise is carried out using a Cox PH model. This will allow the authors to measure the intervention's direct as well as interaction effect and adjust for potential confounding. It is to be noted that balance in baseline factors does not mean that they are auto-adjusted.

Comment 3 – "Due to the relatively young age of participants in ASCEND (mean age = 63.3; SD 9.2) ASCEND-EYE was underpowered to confirm the effects of study treatments on AMD." This is not understandable why due to younger age the study can be underpowered. Power of the study depends on the number of participants and the variance of the outcome variable measured (AMD) in fact. I have noticed in the article (ref no 16) that the sample size was not calculated to estimate the hazard ratio however it is clear from the description of the statistical analysis plan that they considered the time to event as the outcome. Though the hazard ratio was not measured. I, therefore, think that an error is there - an error in sample size calculation or the analysis deviated from the original plan.

Comment 4 - The survival curves were presented without a confidence interval. It is not clear why the authors avoided presenting the uncertainty measure of the survival probability.

Comment 5 – "were analysed using logrank methods." Firstly, the wording needs to be change as "logrank test." My main point is that it is necessary to describe why a logrank test was applied. This is not a good statistical description 'analysed using logrank method' rather I assume you have compared the survival curve using a logrank test. If that is the case, please write that.

**Reviewer**	**2**
**Name**	**von der Burchard, Claus**
**Affiliation**	**Christian Albrechts Universitat zu Kiel, Department of Ophthalmology**
**Date**	**10-Nov-2024**
**COI**	**None**

ASCEND-Eye: Effects of Aspirin and Omega-3 Fatty Acids on Age-Related Macular Degeneration

This is a very well-written study that shows negligible effects of ASS and Omega-3 on AMD development.

The study presents the rationale, statistical methodology and limitations and results in a very structured manner and shows a clear discussion. It should be lauded that the study discusses very openly in how far it is underpowered in many ways:

I am no expert on the statistical analysis but found no weaknesses. Due to the statistical nature of this study, specialist statistical review of the paper should be undertaken.

There is only few changes that I would advise:

- Even though the manuscript states the statistical limitations, this does not become clear in the title; especially, one would think that this was a trial directly designed by ophthalmologists instead of a meta-analysis on diabetic patients. Maybe change it to "ASCEND-Eye: Effects of Aspirin and Omega-3 Fatty Acids on Incidence of Age-Related Macular Degeneration in a Randomized Controlled Trial for Diabetic Patients" or similar

- As mentioned in my suggested title alteration, the study only looks into the incidence of new AMD cases, not the progression of AMD. It is noteworthy that e.g. the AREDS studies found that nutritional supplements might reduce risk of disease progression, especially when stratifying for current AMD status. Similarly, an effect of disease progression might be thinkable. This should be discussed.

- https://pubmed.ncbi.nlm.nih.gov/31358390/ should probably also be included in the discussion section

- The fact that all patients had diabetes, i.e. the effect of aspirin and omega-3 in non-diabetic patients was not studied, should also be highlighted and the potential effects of this be discussed more prominently

---

VERSION 1 - AUTHOR RESPONSE

Reviewer #1

Dr. Mohammed Moinuddin, University of Central Lancashire

The authors presented results from a randomised control trial to assess the effect of Aspirin and Omega-3 fatty acids on age-related macular degeneration. A well-designed RCT with minimised randomisation and masked however the results are negative. It was not an interesting reading due to excessive reference to their other works from the same trial. To me, this piece does not add much value to the science literature as most of the aspects of the

study are already published elsewhere. The only addition is that aspirin and omega-3 fatty acids did not help the patients under study. Moreover, the effect measure and statistical analysis were not up to the mark. My specific observations are below.

<u>Response</u>: *Thank you for taking the time to review our manuscript. The AMD analyses we presented were components of a sub-study of a larger clinical trial. As such, we could not avoid referring to our other work. While previous studies of aspirin and omega-3 fatty acids (FAs) for age-related macular degeneration (AMD) have been conducted, their results have been conflicting. The ASCEND trial afforded an opportunity to contribute further randomized evidence that could be used in future meta-analyses. Our responses to the specific statistical comments follow.*

Comment 1 – I wonder if the outcome AMD was measured from a single eye or both or any? If both eyes were measured there is a possibility of correlated measures. What did the author think about this? Needs clarification.

<u>Response:</u> *As the reviewer has alluded to, AMD is a bilateral disease in which lesions presenting in the left and right eye are usually correlated, whereas most statistical methods assume independence of the sampling units. The AMD diagnoses recorded on ASCEND follow-up forms or the VFQ did not include information about which eye was affected. Therefore, the unit of analysis for the AMD outcome was the participant, and the time-to-first AMD event was calculated as the time to the first post-randomisation report of AMD in either eye. This information has been added to the Methods section (**page 10, lines 239-240; page 11, lines 247-249; and page 11, line 258**).*

Comment 2 – I wonder why the statistical analysis section did not discuss what effect sizes

were and how they were measured. Of course, while I read the results it is visible that the rate ratio was measured. I would suggest describing this in the statistical analysis section. Also, I was wondering why the interaction effect of the two interventions was not measured. It is not even discussed. To me, this analysis of just reporting a rate ratio with a 95% confidence interval is not complete unless a proper modelling exercise is carried out using a Cox PH model. This will allow the authors to measure the intervention's direct as well as interaction effect and adjust for potential confounding. It is to be noted that balance in baseline factors does not mean that they are auto-adjusted.

Response: *It was anticipated that the factorial design of ASCEND would have little or no effect on the statistical sensitivity with which the effects of each treatment arm could be assessed.[1, 2] Moreover, no clinically significant interactions between the study treatments were anticipated.[3] Therefore, Cox modelling was not considered necessary, and the main intention-to-treat effects of treatment were analysed using the log-rank test without allowance for omega-3 FA allocation in comparisons of aspirin versus placebo (and vice versa for omega-3 FAs versus placebo). This information has been added (**page 12, lines 288-291**). However, for completeness, we have added plots of the treatment effects of aspirin stratified by omega-3 FAs (and vice versa) to the **supplemental material (Table S16)**. There is no evidence of heterogeneity. The manuscript has been updated to show that we calculated the average event rate ratio and 95% CI from the logrank test observed minus expected number of events (O-E) and its variance (V); event rate ratio =exp(O-E/V), which gives an estimate of effect size (**page 12, lines 282-288**).*

Comment 3 – "Due to the relatively young age of participants in ASCEND (mean age = 63.3; SD 9.2) ASCEND-EYE was underpowered to confirm the effects of study treatments on AMD." This is not understandable why due to younger age the study can be underpowered. Power of the study depends on the number of participants and the variance of the outcome

variable measured (AMD) in fact. I have noticed in the article (ref no 16) that the sample size was not calculated to estimate the hazard ratio however it is clear from the description of the statistical analysis plan that they considered the time to event as the outcome. Though the hazard ratio was not measured. I, therefore, think that an error is there - an error in sample size calculation or the analysis deviated from the original plan.

Response: *Age-related macular degeneration is a condition that becomes more common with advancing age. In a meta-analysis of studies that used comparable, standardised protocols and comprised data on nearly 25,000 individuals from high-income countries with predominantly white ethnicity, the estimated age-specific prevalence of AMD was less than 0.5% in those aged between 50 and 60, but this increased to 12% and 16% in men and women over 80 years of age, respectively.*[4] The ASCEND population was relatively young (mean age = 63.3; SD 9.2)[5]; therefore, ASCEND-Eye had fewer incident AMD events and thus a lower power to detect a clinically meaningful treatment effect than would be expected in an older population. The sample size could not be increased because ASCEND-Eye was a sub-study of a larger trial. *Calculations were performed to understand the power to detect a clinically meaningful treatment effect on AMD at a two-sided 5% significance level. This information has been added to the Methods section (***pages 11-12, lines 267-276***). The ASCEND-Eye data analysis plan was published on the ASCEND trial website (https://ascend.medsci.ox.ac.uk/) before unblinding the ASCEND-Eye results, and we are pleased to confirm that our analyses are in line with this plan.*

Comment 4 - The survival curves were presented without a confidence interval. It is not clear why the authors avoided presenting the uncertainty measure of the survival probability.

Response: *The survival curves clearly demonstrate no difference in time to AMD diagnosis between the two arms in either randomisation at any time point. Therefore, we feel that adding confidence intervals to the survival plots is unnecessary. We have presented the rate ratios with their 95% confidence intervals, and these confirm that there is no evidence of a difference between the arms.*

Comment 5 – “were analysed using logrank methods.” Firstly, the wording needs to be change as “logrank test.” My main point is that it is necessary to describe why a logrank test was applied. This is not a good statistical description 'analysed using logrank method' rather I assume you have compared the survival curve using a logrank test. If that is the case, please write that.

Response: *This is addressed in our response to comment #2.*

Reviewer#2

Dr. Claus von der Burchard, Christian Albrechts Universitat zu Kiel

Comments to the Author:

This is a very well-written study that shows negligible effects of ASS and Omega-3 on AMD development. The study presents the rationale, statistical methodology and limitations and results in a very structured manner and shows a clear discussion. It should be lauded that the study discusses very openly in how far it is underpowered in many ways.

I am no expert on the statistical analysis but found no weaknesses. Due to the statistical nature of this study, specialist statistical review of the paper should be undertaken.

There is only few changes that I would advise:

- Even though the manuscript states the statistical limitations, this does not become clear in the title; especially, one would think that this was a trial directly designed by ophthalmologists instead of a meta-analysis on diabetic patients. Maybe change it to “ASCEND-Eye: Effects of Aspirin and Omega-3 Fatty Acids on Incidence of Age-Related Macular Degeneration in a Randomized Controlled Trial for Diabetic Patients” or similar

__Response__: *Thank you for your positive comments regarding our manuscript. This was not a meta-analysis. It is a report of a sub-study of a large randomised cardiovascular trial in diabetes. In these analyses, the effects of aspirin and omega-3 FAs on AMD are reported. We have changed the title in accordance with the editorial requirements (**Page 1, lines 1-3**).*

- As mentioned in my suggested title alteration, the study only looks into the incidence of new AMD cases, not the progression of AMD. It is noteworthy that e.g. the AREDS studies found that nutritional supplements might reduce risk of disease progression, especially when stratifying for current AMD status. Similarly, an effect of disease progression might be thinkable. This should be discussed.

__Response:__ *On **page 19, lines 472-474,** we explain that ASCEND-Eye was limited by a lack of retinal imaging, which is necessary for detailed phenotyping of early vs late AMD and wet or dry subtypes. We have added a comment to the Discussion to indicate that since the AMD status of participants at baseline was unknown, we could not ascertain their disease progression or assess any treatment effects on disease progression (**page 19, lines 474-477**). However, randomization ensures that these factors should be balanced across treatment*

groups and not introduce bias in the comparisons. It also seems unlikely that a treatment may have a large effect on progression and no effect on incidence.

- https://pubmed.ncbi.nlm.nih.gov/31358390/ should probably also be included in the discussion section

Response: *Our discussion focuses on randomized trial evidence or aspirin and, separately omega-3 FAs for AMD. The suggested paper describes observational analyses of associations between aspirin use and AMD progression among participants of the AREDS and AREDS2 trials of antioxidant multivitamins. Given its observational design, the results may be confounded by variables not accounted for in the analyses. Therefore, this reference has not been added.*

- The fact that all patients had diabetes, i.e. the effect of aspirin and omega-3 in non-diabetic patients was not studied, should also be highlighted and the potential effects of this be discussed more prominently.

Response: *We have updated the final paragraph of the Introduction section (**Page 9, lines 205-207**). Diabetes and AMD are not considered to be pathologically related to each other.[6-8]*

1. Peto R, Pike MC, et al. Design and analysis of randomized clinical trials requiring prolonged observation of each patient. I. Introduction and design. British Journal of Cancer. 1976;34(6):585-612.
2. Peto R, Pike MC, et al. Design and analysis of randomized clinical trials requiring prolonged observation of each patient. II. analysis and examples. British Journal of Cancer. 1977;35(1):1-39.

3. Sapieha P, Stahl A, et al. 5-Lipoxygenase Metabolite 4-HDHA Is a Mediator of the Antiangiogenic Effect of ω-3 Polyunsaturated Fatty Acids. Science Translational Medicine. 2011;3(69):69ra12-69ra12.
4. Friedman DS, O'Colmain BJ, et al. Prevalence of age-related macular degeneration in the United States. Arch Ophthalmol. 2004;122(4):564-572.
5. Bowman L, Mafham M, et al. ASCEND: A Study of Cardiovascular Events iN Diabetes: Characteristics of a randomized trial of aspirin and of omega-3 fatty acid supplementation in 15,480 people with diabetes. American Heart Journal. 2018;198:135-144.
6. Coleman HR, Chan C-C, et al. Age-related macular degeneration. The Lancet. 2008;372(9652):1835-1845.
7. Klein R. Early Age-related Maculopathy in the Cardiovascular Health Study. Ophthalmology. 2003;110:25-33.
8. The Atherosclerosis Risk in Communities Study Investigators. Prevalence of age-related maculopathy in the Atherosclerosis Risk in Communities Study. Archives of Ophthalmology. 1999;117:1203-1210.

VERSION 2 - REVIEW

**Reviewer**	**1**
**Name**	**Moinuddin, Mohammed**
**Affiliation**	**University of Central Lancashire, School of Medicine and Dentistry**
**Date**	**23-Dec-2024**
**COI**	

I thank the authors for clarifying and justifying their statistical analysis in the method section. Unfortunately, the study is severely underpowered (power = 24-41%), which means a very high type-II error probability. With this power, the tests applied could not detect whether there was any significant difference. So, the conclusion lost its validity. The ‘null-effect’ of intervention is not the main issue; the issue is the uncertainty of the ‘null-effect’ of the intervention.

**Reviewer**	**2**
**Name**	**von der Burchard, Claus**
**Affiliation**	**Christian Albrechts Universitat zu Kiel, Department of Ophthalmology**
**Date**	**19-Dec-2024**

COI

From my side, the authors have satisfactorily addressed all comments by the reviewers.
